# Burden of women’s cancers in the group of twenty (G20) from 1990 to 2023: epidemiological trends and impact from fertility, quality of care, and survival

**DOI:** 10.1016/j.mmr.2026.100026

**Published:** 2026-04-27

**Authors:** Meng-Long Li, Rui-Shu Tang, Nan Wang, Jin-Lei Qi, Hui-Ming He, Meng-Ying Guan, Miao Li, Bing-Qing Wu, Yeerlin Asihaer, Sten H. Vermund, Yi-Fei Hu

**Affiliations:** a Department of Child, Adolescent Health and Maternal Care, School of Public Health, Capital Medical University, Beijing 100069, China; b Evidence-based Medicine Center, Beijing Luhe Hospital, Capital Medical University, Beijing 101149, China; c Department of Gynecology, Beijing Youan Hospital, Capital Medical University, Beijing 100069, China; d National Center for Chronic and Noncommunicable Disease Control and Prevention, Chinese Center for Disease Control and Prevention (China CDC), Beijing 100050, China; e College of Public Health, University of South Florida, Tampa, FL 33612, USA; f Beijing Key Laboratory of Environment and Aging, Capital Medical University, Beijing 100069, China

**Keywords:** Women’s cancers, Global burden of diseases, Survival, Dominance analysis, Fertility

## Abstract

**Background:**

Cancer in women represents a significant disease burden, posing challenges for prevention, treatment, and caregiving. This study aimed to analyze the epidemiological trends of the women’s cancer burden and the main influencing factors in the group of twenty (G20) from 1990 to 2023.

**Methods:**

Incidence, prevalence, mortality, and disability-adjusted life years (DALYs) for breast, cervical, uterine, and ovarian cancers, as well as fertility rates for G20 and its 98 locations, were sourced from the Global Burden of Disease Study 2023. Age-standardized rates (ASRs), quality of care index (QCI), and 5-year relative survival of integrated women’s cancers were calculated. Average annual percent changes (AAPCs) were used to determine the temporal trends by age and region. Decomposition analysis identified drivers of changes in case numbers, linear regression assessed the associations with DALY rate changes, and dominance analysis identified dominant predictors.

**Results:**

In 2023, the incidence, prevalence, mortality, and DALYs from women’s cancers in G20 were 3.29 [95% uncertainty interval (UI) 2.60−4.14], 26.71 (95% UI 21.99−32.40), 1.16 (95% UI 0.91−1.45), and 36.58 million (95% UI 28.40−46.32), respectively, with ASRs of 87.63/100,000 (95% UI 65.12−115.85), 706.16/100,000 (95% UI 555.75−890.02), 30.03/100,000 (95% UI 22.10−39.58), and 994.79/100,000 (95% UI 728.43−1328.81). The QCI was 75.13 [95% confidence interval (CI) 73.67−76.59], and the 5-year relative survival was 65.74% (95% CI 65.53−65.95). From 1990 to 2023, there was a significant increase in incidence, prevalence, mortality, and DALYs in G20, primarily driven by population growth. Age-standardized incidence rate, QCI, and 5-year relative survival increased, while age-standardized mortality and DALY rates decreased. Changes in prevalence rates of breast cancer and cervical cancer for women aged 15−49 years were positively associated with changes in DALY rates of women’s cancers, whereas changes in the total fertility rate were negatively associated. Dominance analysis confirmed these three factors consistently as dominant predictors between 1990 and 2023. Reducing the prevalence of breast and cervical cancers and increasing fertility among women aged 15−49 years could lower the overall DALY burden attributable to women’s cancer.

**Conclusions:**

The incidence, prevalence, mortality, and DALYs of women’s cancers in G20 have increased substantially from 1990 to 2023. Tailored prevention strategies should consider age and cancer type, emphasizing reproductive health for women of reproductive age.

## Background

According to global cancer statistics, cancer accounted for 8.99 million and 9.66 million new cases and 4.61 million and 4.31 million deaths among women in 2023 and 2022, respectively, as reported by the Global Burden of Disease Study and Global Cancer Observatory [1], [2]. Breast cancer, cervical cancer, ovarian cancer, and uterine cancer collectively accounted for 38.3% of new cases and 30.7% of deaths among all cancers in women, highlighting significant challenges in cancer prevention, treatment, and caregiving due to social inequities related to women [2], [3]. As one of the most important gatherings of the world’s economic leaders, the Group of Twenty (G20) includes 19 countries and the European Union and the African Union, representing two-thirds of the global population. Crucially, the G20 encompasses a diverse spectrum of socioeconomic development levels, ranging from high-income to lower-middle-income economies with significant health disparities in maternal and child health and the burden of non-communicable diseases [4], [5], [6]. This unique composition makes it an ideal group for assessing health trends, offering insights that are highly representative of the world’s varied economic and health landscapes. Hence, updates on epidemiological trends of women’s cancers are needed to inform interventions in the G20.

Breast, cervical, and ovarian cancer collectively impose a significant health burden, accounting for approximately 3.50 million new cases and 1.37 million deaths globally in 2023 [1], impacting millions of women and their families annually [2], [3]. Among these, breast cancer and cervical cancer are the first and fourth most common cancers in women, respectively, and contributed to 468,000 new maternal orphans in 2020 [2], [7], [8], [9]. Ovarian cancer is a top-10 cause of cancer-related death, with a rising mortality due to its asymptomatic nature and late detection. The disease burden associated with these major women’s cancers is exacerbated by population growth, revealing stark disparities across geographical regions and nations [3], [10], [11]. These disparities stem from differences in socioeconomic development, which in turn affect the improvement of health systems in prevention, treatment, quality of care, and survival [3], [8], [10], [12]. To evaluate these performance gaps, the quality of care index (QCI) has been introduced to evaluate healthcare system disparities across regions [8], [13], [14]. Previous studies have indicated an association between hormonal exposure, reproductive behaviors, and women’s cancers [15], [16], [17]. Moreover, previous studies have mainly focused on specific cancer types, age groups, or limited measures, overlooking a comprehensive assessment of the factors contributing to women’s cancer burden [8], [10], [15].

Utilizing the updated Global Burden of Diseases, Injuries, and Risk Factors Study (GBD) 2023 data, this study conducts a comprehensive analysis of the burden of integrated women’s cancer and presents epidemiological temporal trends for women’s cancers from 1990 to 2023 in the G20 and its 98 locations. Given the decline in fertility across the world [18], this study posits a potential association between the fertility rate and women’s cancer burden and explores the implications of fertility changes on the burden of women’s cancer. Furthermore, this study examines the dominant predictors of change in overall disability-adjusted life year (DALY) rates, including changes in the prevalence of 4 cancer types and fertility among women aged 15−49 years, population aging, population growth, socio-demographic index (SDI), quality of care, and survival rate. The study aims to inform healthcare policy improvements focused on women of reproductive age across various countries and territories.

## Methods

### Data source

The GBD study is a comprehensive epidemiological study that provides standardized estimates of the burden of 463 health outcomes and risk factors in 204 countries and territories [1], [19], [20], [21], [22], [23]. Major measures include incidence, mortality, DALYs, prevalence, years of life lost (YLLs), and years lived with disability (YLDs) worldwide. Our study used these data to focus on 4 women’s cancers statistics: breast cancer [International Classification of Diseases, 10th revision (ICD-10): C50−C50.9, D05−D05.9, D24−D24.9, D48.6, D49.3], cervical cancer (ICD-10: C53−C53.9, D06−D06.9, D26.0), ovarian cancer (ICD-10: C56−C56.9, D27−D27.9, D39.1), and uterine cancer (ICD-10: C54−C54.9, D07.0−D07.2, D26.1−D26.9).

Data on the number and rate of 6 measures, incidence, mortality, DALYs, prevalence, YLLs, and YLDs, for the 4 types from 1990 to 2023 were extracted for 280 geographical units (level 3 and above) and all age groups by 5-year interval, and included age-specific fertility rates and SDI. The SDI served as a composite indicator to measure the development status of a country or territory, integrating income per capita, average educational attainment, and fertility rates. Geographical stratification was applied to the G20 and its 98 locations. Age stratifications were categorized into three groups: 15−49 years, 50−69 years, and ≥70 years. Total fertility rate in this study was calculated by summing the age-specific fertility rates of 7 age groups from 15−19 years to 45−49 years.

Both incidence and prevalence were modeled using the Bayesian Meta-regression tool, Disease Modelling Meta-Regression, version 2.1 (DisMod-MR 2.1), and mortality was estimated using the Cause of Death Ensemble model (CODEm). YLLs were calculated by multiplying the number of deaths by the remaining life expectancy at the age of death for each cause. YLDs were derived from the prevalence of health conditions multiplied by their disability weights. DALYs were the sum of YLLs and YLDs by location, age, sex, year, and cause, representing the total healthy years lost from disease onset to death [12], [19], [20]. Age-specific fertility rates for 5-year age groups were generated using mixed-effects regression models and spatiotemporal Gaussian process regression [18].

### Statistical analysis

Age-standardized rates (ASRs) for incidence, prevalence, mortality, DALYs, YLLs, and YLDs are used to minimize the influence of demographic structures and enable comparisons across time and locations. ASRs for overall women and three age groups were calculated using the formula: $\frac{\sum_{\text{i}=1}^{N}a_{i}w_{i}}{\sum_{\text{i}=1}^{N}w_{i}}\times 100,000$, where $a_{i}$ is the age-specific rate in the $i^{\text{th}}$ age group, $w_{i}$ is the weight of the corresponding GBD standard population to align with the procedure of GBD estimates, and N is the number of age groups. The 95% uncertainty intervals (UIs) were obtained using the lower and upper bounds via the same procedure [10], [21].

Four secondary indices associated with quality of care, mortality-to-incidence ratio (MIR), YLL-to-YLD ratio, DALY-to-prevalence ratio, and prevalence-to-incidence ratio were generated based on the ASRs of 6 primary measures [8], [14]. These indices were then transformed into a smaller set of principal components using principal component analysis across 280 locations from 1990 to 2023. The leading component was rescaled as the QCI, scored from 0 to 100, where higher QCI scores indicate better quality of care. The 5-year relative survival was estimated using the formula (1−MIR) at regional and national levels from 1990 to 2023 [24], [25] as a percentage ranging from 0% to 100%, with higher percentages indicating better survival in healthcare systems.

The average annual percent changes (AAPCs) were used to identify temporal trends in age-standardized incidence, prevalence, mortality, and DALY rates, as well as QCI and 5-year relative survival. AAPCs with 95% confident intervals (CIs) were generated from the log-linear Joinpoint regression model using the formula: $[\exp\left(\frac{\sum w_{i}\beta_{i}}{\sum w_{i}}\right)-1]\times 100\%$, where i is the segment in the desired range of calendar years, $w_{i}$ is the years of the corresponding segment and $\beta_{i}$ is the slope coefficient for the i^th^ segment [26], [27], [28], [29]. An increasing trend is defined when both the AAPC and its lower 95% CI are >0. A decreasing trend is defined when both the AAPC and its upper 95% CI are<0. If the 95% CI includes 0, the trend is considered relatively stable.

To identify the key drivers of changes in the number of women’s cancer cases from 1990 to 2023, a decomposition analysis was conducted using Gupta’s methodology [28], [29], [30]. In short, the individual influences of population growth, population aging, and epidemiological change in age-specific rates on the burden of women’s cancers, including incidence, prevalence, mortality, and DALYs, were assessed in G20.

Linear regression models were employed to assess associations of 10 predictors with changes in DALY rates for women in G20 and its 98 locations from 1990 to 2023. The predictors included changes in prevalence rates for breast, cervical, uterine, and ovarian cancers in women aged 15−49 years; changes in total fertility rate of women aged 15−49 years; changes in population proportions of women aged 60 years and above; changes in QCI; changes in 5-year relative survival; changes in SDI; and changes in the total female population. Percent changes for these predictors and DALY rates between specific years and 1990 were calculated using the formula: $\text{percent changes}=\frac{\text{values in specific year}-\text{values in }1990}{\text{values in }1990}\times 100\%$. Since these indicators were calculated as percentage changes, placing the indicators under the same comparable framework, a normal distribution was assumed, and potential linear associations were determined using linear regression. To further assess the contribution of predictors, a dominance analysis was conducted after the linear regression. This method decomposes the variance explained by each predictor across all possible models to address multicollinearity. Thus, the relative importance of variables can be determined by comparing the average *R*² (dominance) values across all models [31], [32].

All data management, statistical analysis, and graphical representations were conducted using Microsoft Excel (Microsoft Corp., Washington, DC, USA), Statistical Analysis System® (version 9.4, SAS Institute Inc., Cary, NC, USA), Joinpoint Regression Program (version 5.0.2, the National Cancer Institute, Rockville, MD, USA), and R software (version 4.3.2, R Foundation, Vienna, Austria).

## Results

### Women’s cancers statistics in 2023 and trends from 1990 to 2023 in G20

In 2023, the incidence of women’s cancers in G20 was 3.29 million (95% UI 2.60−4.14), with 1.16 million mortality (95% UI 0.91−1.45). The corresponding age-standardized incidence and mortality rates were 87.63/100,000 (95% UI 65.12−115.85) and 30.03/100,000 (95% UI 22.10−39.58), respectively. The QCI was 75.13 (95% CI 73.67−76.59), and the 5-year relative survival was 65.74% (95% CI 65.53−65.95) **(**Table 1**)**. Meanwhile, there were an estimated 26.71 million prevalence [(95% UI 21.99−32.40); ASR 706.16/100,000 (95% UI 555.75−890.02)] and 36.58 million DALYs [(95% UI 28.40−46.32); ASR 994.79/100,000 (95% UI 728.43−1328.81)] attributed to women’s cancers **(**Table 1**;** Additional file 1**:** Table S1**)**. Increasing trends in age-standardized incidence rate (AAPC=0.21, 95% CI 0.07−0.34), QCI (AAPC=0.27, 95% CI 0.22−0.33), and the 5-year relative survival (AAPC=0.41, 95% CI 0.37−0.44) were observed for women’s cancers from 1990 to 2023, while age-standardized mortality (AAPC=−0.45, 95% CI −0.57 to −0.34) and DALY rates (AAPC=−0.28, 95% CI −0.38 to −0.19) declined **(**Table 1**)**.

**Table 1:** Incidence, mortality, DALY, QCI, and 5-year relative survival of women’s cancers by type and age in 2023 and trends from 1990 to 2023 in G20.

**Characteristic**	**Incidence**			**Mortality**			**DALY**			**QCI**		**5*****-*****year relative survival**	
	**Number** **(million,** **95% UI)**	**ASR** **(/100,000,** **95% UI)**	**AAPC** **(95% CI)**	**Number** **(million,** **95% UI)**	**ASR** **(/100,000, 95% UI)**	**AAPC** **(95% CI)**	**Number** **(million, 95% UI)**	**ASR** **(/100,000, 95% UI)**	**AAPC** **(95% CI)**	**Estimate** **(95% CI)**	**AAPC** **(95% CI)**	**Rate** **(%, 95% CI)**	**AAPC** **(95% CI)**
Women’s cancer	3.29 (2.60−4.14)	87.63 (65.12−115.85)	0.21 (0.07−0.34)	1.16 (0.91−1.45)	30.03 (22.10−39.58)	*−*0.45 (*−*0.57 to *−*0.34)	36.58 (28.40−46.32)	994.79 (728.43−1328.81)	*−*0.28 (*−*0.38 to *−*0.19)	75.13 (73.67−76.59)	0.27 (0.22−0.33)	65.74 (65.53−65.95)	0.41 (0.37−0.44)
Type													
Breast cancer	1.89 (1.54−2.26)	49.59 (38.20−62.76)	0.31 (0.18−0.44)	0.61 (0.49−0.73)	15.58 (11.86−19.67)	*−*0.32 (*−*0.45 to *−*0.19)	18.79 (15.19−22.62)	507.78 (386.35−646.93)	*−*0.15 (*−*0.25 to *−*0.04)	80.07 (76.92−83.23)	0.06 (0.02−0.10)	68.58 (68.35−68.80)	0.34 (0.32−0.36)
Cervical cancer	0.70 (0.50−0.97)	19.91 (13.24−29.00)	*−*0.02 (*−*0.11 to 0.07)	0.29 (0.21−0.40)	7.88 (5.31−11.37)	*−*0.57 (*−*0.72 to *−*0.42)	10.55 (7.50−14.62)	297.03 (200.76−430.11)	*−*0.36 (*−*0.49 to *−*0.23)	61.78 (60.29−63.27)	0.62 (0.56−0.68)	60.44 (60.01−60.87)	0.42 (0.39−0.46)
Uterine cancer	0.44 (0.35−0.57)	11.15 (8.50−14.93)	0.44 (0.32−0.57)	0.09 (0.07−0.11)	2.14 (1.61−2.84)	*−*0.78 (*−*0.96 to *−*0.59)	2.29 (1.80−2.99)	58.39 (43.66−78.89)	*−*0.78 (*−*0.92 to *−*0.64)	86.26 (85.71−86.82)	0.55 (0.51−0.59)	80.77 (80.52−81.02)	0.38 (0.36−0.40)
Ovarian cancer	0.26 (0.21−0.32)	6.97 (5.19−9.15)	*−*0.22 (*−*0.31 to *−*0.14)	0.17 (0.14−0.21)	4.42 (3.32−5.71)	*−*0.48 (*−*0.60 to *−*0.36)	4.95 (3.90−6.10)	131.60 (97.66−172.88)	*−*0.34 (*−*0.44 to *−*0.24)	63.19 (54.36−72.01)	0.56 (0.48−0.65)	36.62 (35.80−37.45)	0.51 (0.46−0.56)
Age group (years)													
15−49	1.05 (0.75−1.46)	65.74 (46.70−91.23)	0.54 (0.48−0.60)	0.28 (0.20−0.39)	17.35 (12.30−23.92)	0.17 (0.07−0.28)	14.54 (10.32−20.04)	905.09 (641.23−1249.73)	0.21 (0.10−0.32)	71.25 (58.63−83.87)	0.14 (0.08−0.19)	73.61 (73.49−73.72)	0.14 (0.12−0.16)
50−69	1.46 (1.11−1.89)	233.63 (177.75−303.97)	0.04 (*−*0.06 to 0.15)	0.48 (0.36−0.63)	76.36 (56.88−100.79)	*−*0.69 (*−*0.81 to *−*0.57)	15.68 (11.73−20.61)	2523.95 (1887.08−3319.41)	*−*0.64 (*−*0.76 to *−*0.51)	78.14 (76.64−79.64)	0.33 (0.29−0.37)	67.32 (66.74−67.89)	0.45 (0.42−0.49)
≥ 70	0.79 (0.60−0.98)	309.68 (236.81−387.14)	*−*0.06 (*−*0.14 to 0.03)	0.40 (0.30−0.51)	158.23 (118.60−199.60)	*−*0.62 (*−*0.83 to *−*0.42)	6.36 (4.85−8.00)	2498.72 (1903.58−3141.77)	*−*0.63 (*−*0.83 to *−*0.42)	79.07 (75.66−82.48)	0.20 (0.17−0.23)	48.91 (48.17−49.64)	0.79 (0.72−0.86)

The AAPC reflects trends in ASR, QCI, and 5-year relative survival rate from 1990 to 2023, while all other data pertain to 2023. Women’s cancer refers to a combination of breast cancer, cervical cancer, uterine cancer, and ovarian cancer; AAPC. Average annual percent change; ASR. Age-standardized rate; DALY. Disability-adjusted life year; QCI. Quality of care index; UI. Uncertainty interval; CI. Confidence interval

In 2023, breast cancer had the highest burden among women’s cancers, with 18.79 million (95% UI 15.19−22.62) DALYs, followed by cervical cancer [10.55 million (95% UI 7.50−14.62) DALYs], ovarian cancer [4.95 million (95% UI 3.90−6.10) DALYs], and uterine cancer [2.29 million (95% UI 1.80−2.99) DALYs] **(**Table 1**)**. Uterine cancer presented a higher incidence and prevalence but lower DALYs with the highest QCI [86.26 (95% CI 85.71−86.82)] and 5-year relative survival [80.77% (95% CI 80.52−81.02)] than ovarian cancer **(**Table 1**;** Additional file 1**:** Table S1**)**. The age-standardized incidence rates showed an increasing trend for breast cancer (AAPC=0.31, 95% CI 0.18−0.44) and uterine cancer (AAPC=0.44, 95% CI 0.32−0.57), a stable trend for cervical cancer (AAPC=−0.02, 95% CI −0.11 to 0.07), and a decreasing trend for ovarian cancer (AAPC=−0.22, 95% CI -0.31 to -0.14) **(**Table 1**)**. All 4 women’s cancer types presented decreasing trends in age-standardized mortality and DALY (all AAPCs<0), with corresponding increases in QCI and 5-year relative survival from 1990 to 2023 (all AAPCs>0) **(**Table 1**)**.

In 2023, there were 1.05 million incidences of women’s cancers in the reproductive age group (15−49 years), with a 0.72-fold and 1.33-fold number of incidences in women aged 50−69 years (1.05 million vs. 1.46 million) and ≥70 years (1.05 million vs. 0.79 million), respectively **(**Table 1**)**. Women aged 15−49 years bore a higher DALYs ratio, 0.93-fold and 2.29-fold compared to 50−69 years (14.54 million vs. 15.68 million) and ≥70 years (14.54 million vs. 6.36 million), respectively **(**Table 1**)**. Increasing trends in age-standardized incidence, mortality, and DALY rates, QCI, and 5-year relative survival (all AAPCs>0) were observed in women aged 15−49 years. Consistent with the findings in all women, increasing trends in QCI and 5-year relative survival (all AAPCs>0), with decreasing trends in age-standardized mortality and DALY rates (all AAPCs<0), were observed in women aged 50−69 years and ≥70 years from 1990 to 2023 **(**Table 1**)**. Detailed women’s cancer statistics and trends by location, cancer type, and age are presented in Additional file 1**:** Tables S2-S4 and Figs. S1-S7.

### Changes in women’s cancer cases and their driving components in G20

**Fig. 1:**
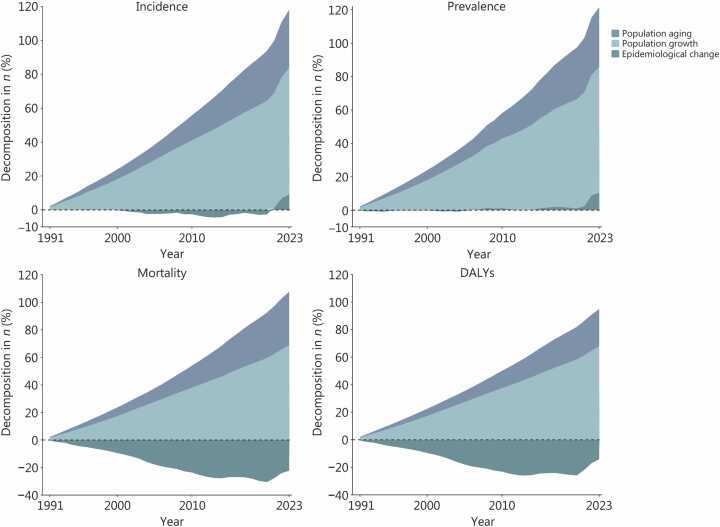
**Decomposition analysis of changes in incidence, prevalence, mortality, and DALYs of women’s cancers in G20 from 1991 to 2023, compared with 1990**. DALY. Disability-adjusted life year.

From 1990 to 2023, the incidence, prevalence, mortality, and DALYs of women’s cancers in G20 increased significantly by 117.95%, 121.48%, 85.25%, and 80.80%, respectively (Fig. 1**;** Additional file 1**:** Fig. S3). The highest increases in overall percent change were observed in Saudi Arabia in incidence (626.90%), prevalence (618.44%), while South Africa showed the highest increase in overall percent change in mortality (370.75%) and DALYs (387.57%). In contrast, the United Kingdom showed the lowest change in overall incidence (21.48%), prevalence (27.10%), mortality (−5.72%), and DALYs (−17.57%) **(**Additional file 1**:** Table S5 and Fig. S8**)**. For women aged 15−49 years, relatively lower increases in overall percent change in incidence (99.84%) and prevalence (99.85%) of women’s cancers were observed compared to women aged 50−69 years and ≥70 years **(**Additional file 1**:** Fig. S3**)**.

Decomposition analysis by year suggested that population growth was the primary contributor to changes in incidence, prevalence, mortality, and DALYs of women’s cancers from 1990 to 2023 in G20, followed by population aging. Epidemiological changes showed an inverse pattern for mortality and DALYs (Fig. 1**)**. Specifically, population growth contributed 74.67%, 75.36%, 68.94%, and 67.89% to the overall changes in incidence, prevalence, mortality, and DALYs, respectively. Population aging accounted for 34.08%, 35.76%, 38.64%, and 27.08%, while epidemiological changes contributed 9.20%, 10.35%, −22.33%, and −14.17% to these outcomes **(**Additional file 1**:** Table S5 and Fig. S8**)**.

The relative contributions of these components varied across locations. Population aging was the dominant contributor in the Republic of Korea (accounting for 90.54%, 105.48%, and 96.01% of changes in incidence, prevalence, and mortality, respectively) and in Indonesia (accounting for 61.55% of changes in DALYs). Population growth contributed substantially in Saudi Arabia (266.21% and 263.66% of changes in incidence and prevalence) and the African Union (196.18% and 194.24% of changes in mortality and DALYs). Epidemiological changes also contributed to observed patterns in Saudi Arabia (297.45% and 289.81% of changes in incidence and prevalence) and South Africa (145.99% and 161.18% of changes in mortality and DALYs) **(**Additional file 1**:** Table S5 and Fig. S8**)**.

### Country-level women’s cancers statistics by age in 2023

**Fig. 2:**
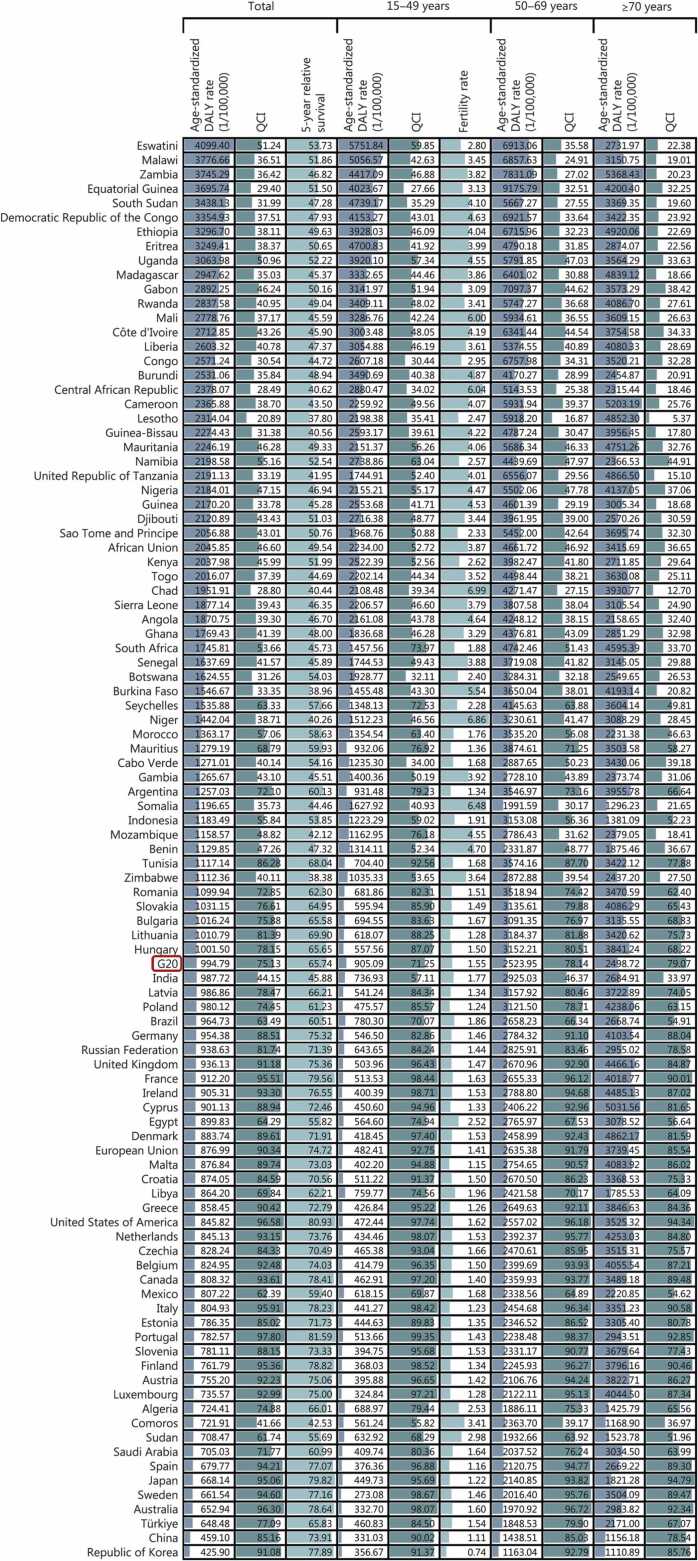
**Age-standardized DALY rate, QCI, 5-year relative survival of women’s cancers, and total fertility rate by age in G20 and its 98 locations in 2023**. DALY. Disability-adjusted life year; QCI. Quality of care index.

Age-standardized DALY rate, QCI, and 5-year relative survival of women’s cancers by location and age in 2023 in G20 are presented in Fig. 2. In 2023, a higher age-standardized DALY rate than the G20 average (994.79/100,000) was noted in 57 locations for women’s cancers. Stratified by ages 15−49 years, 50−69 years, and ≥70 years, 51 (52.04%), 70 (71.43%), and 78 (79.59%) locations exceeded the G20 average, respectively. Specifically, the highest age-standardized DALY rates across the three age groups were observed in Eswatini (5751.84/100,000 in women aged 15−49 years), Equatorial Guinea (9175.79/100,000 in women aged 50−69 years), and Zambia (5368.43/100,000 in women aged ≥70 years). Conversely, the lowest age-standardized DALY rates were recorded in Sweden (273.08/100,000 in women aged 15−49 years) and the Republic of Korea (1163.04/100,000 in women aged 50−69 years, 1110.89/100,000 in women aged ≥70 years) **(**Fig. 2**)**.

In 2023, the QCI for women’s cancers varied substantially across locations, ranging from 20.89 (Lesotho) to 97.80 (Portugal) for all women; from 27.66 (Equatorial Guinea) to 99.35 (Portugal) among women aged 15−49 years; from 16.87 (Lesotho) to 98.37 (Portugal) among those aged 50−69 years; and from 5.37 (Lesotho) to 94.79 (Japan) among women aged ≥70 years. A total of 36 (36.73%), 47 (47.96%), 36 (36.73%), and 24 (24.49%) locations had a QCI value above the G20 average in these respective age groups **(**Fig. 2**)**. Locations with QCI values exceeding the G20 average generally showed higher 5-year relative survivals. Overall, 5-year relative survival for women’s cancers ranged from 37.80% (Lesotho) to 81.59% (Portugal). In 2023, the total fertility rate among women aged 15−49 years in the G20 was 1.55, ranging from 0.74 (Republic of Korea) to 6.99 (Chad). A total of 64 (65.31%) locations had fertility rates above the G20 average **(**Fig. 2**)**.

### Associations and dominant predictors for change in DALY rates for women’s cancers

**Fig. 3:**
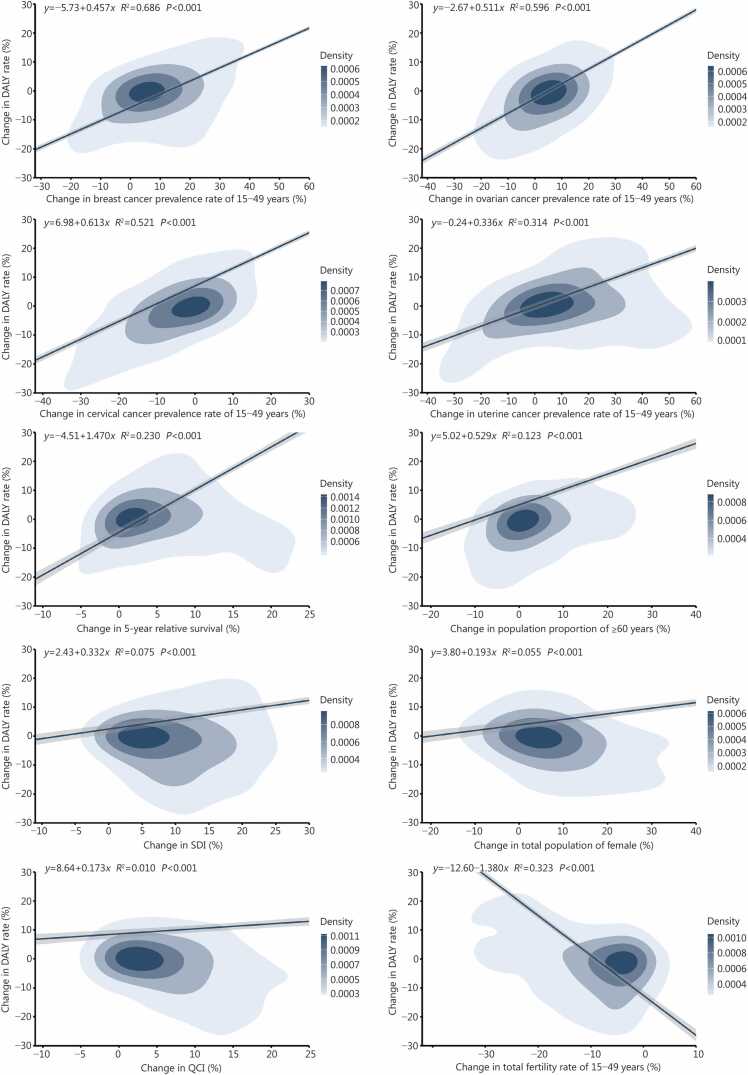
**Linear associations of change in prevalence rates of 4 cancer types, 5-year relative survival, population aging, SDI, female population, QCI, and fertility rates with change in DALY rates of women’s cancers in G20 and its 98 locations from 1991 to 2023**. DALY. Disability-adjusted life year; SDI. Socio-demographic index; QCI. Quality of care index.

Positive associations were noted between changes in the prevalence rates of all 4 cancer types among women aged 15−49 years and changes in the DALY rate for women’s cancers across the total female population from 1991 to 2023. Significant coefficients of determination were observed for prevalence rates of women aged 15−49 years and changes in DALY rate for women’s cancers, respectively, *R*^2^=0.686 and *β* = 0.457 for breast cancer, *R*^2^=0.596 and *β*=0.511 for ovarian cancer, *R*^2^=0.521 and *β*=0.613 for cervical cancer, *R*^2^=0.314 and *β*=0.336 for uterine cancer, all *P*-values*<*0.001. Negative associations were found between the changes in DALY rate of women’s cancers and changes in total fertility rates for women ages 15−49 years (*R*^2^=0.323 and *β*=−1.380, *P<*0.001) **(**Fig. 3**)**. The percent changes of the indicators in G20 and its 98 locations from the 1990 baseline are summarized in Additional file 1**:** Table S6.

Dominance analysis showed that changes in breast cancer prevalence rate (29.52%), ovarian cancer prevalence rate (22.85%), cervical cancer prevalence rate (17.12%), uterine cancer prevalence rate (9.17%), and total fertility rate (8.91%) of women aged 15−49 years were the top 5 dominant factors for the changes in DALY rates of women’s cancers in 2023. Compared to 2010 and 2000, the dominance of changes in total fertility rates of women aged 15−49 years changed, from 7.35% in 2000 to 12.05% in 2010. An upward trend was observed for ovarian cancer prevalence in this age group, rising from 4.69% in 2000 to 17.83% in 2010. In contrast, the dominance of changes in cervical cancer prevalence rates of women aged 15−49 years declined significantly, from 32.51% in 2000 to 24.70% in 2010. From 1991 to 2023, the 5 dominance factors accounted for 82.87% of the overall interpretable changes in DALY rates of women’s cancers, namely changes in breast cancer, ovarian cancer, cervical cancer, and uterine cancer prevalence rates, and total fertility rates of women aged 15−49 years (all *P*-values<0.001) **(**Table 2**;** Additional file 1**:** Table S7**)**.

**Table 2:** Dominance analysis of all predictors for the change in DALY rates of women’s cancers in G20 and its 98 locations, compared with 1990.

**Predictors**	**2023 (*****n*****=99)**			**Dominance [*****R***^**2**^**(%)]**		
	**Percent change** **[% (SE)]**	***β***	**Dominance** **[*****R***^**2**^**(%)]**	**2010 (*****n*****=99)**	**2000 (*****n*****=99)**	**1991–2023(*****n*****=3267)**
Percent change in breast cancer prevalence rate of 15−49 years	116.69 (11.41)	0.336	0.267 (29.52)⁎	0.210 (25.16)⁎	0.138 (15.26)⁎	0.236 (27.00)⁎
Percent change in ovarian cancer prevalence rate of 15−49 years	82.35 (10.04)	0.124	0.207 (22.85)⁎	0.149 (17.83)⁎	0.042 (4.69)	0.165 (18.84)⁎
Percent change in cervical cancer prevalence rate of 15−49 years	35.03 (7.01)	0.201	0.155 (17.12)⁎	0.206 (24.70)⁎	0.293 (32.51)⁎	0.180 (20.62)⁎
Percent change in uterine cancer prevalence rate of 15−49 years	105.00 (8.37)	*−*0.008	0.083 (9.17)	0.039 (4.68)	0.050 (5.52)	0.062 (7.04)⁎
Percent change in total fertility rate of 15−49 years	*−*31.54 (1.69)	*−*0.456	0.081 (8.91)⁎	0.100 (12.05)⁎	0.066 (7.35)⁎	0.082 (9.37)⁎
Percent change in 5*-*year relative survival	22.58 (1.29)	*−*0.401	0.034 (3.75)	0.030 (3.59)	0.058 (6.47)	0.043 (4.97)⁎
Percent change in quality of care index	28.47 (2.06)	*−*0.680	0.031 (3.42)⁎	0.075 (8.94)⁎	0.214 (23.78)⁎	0.056 (6.36)⁎
Percent change in total female population	78.66 (7.08)	0.057	0.025 (2.72)	0.004 (0.46)	0.011 (1.19)⁎	0.013 (1.45)⁎
Percent change in socio*−*demographic index	52.76 (4.79)	0.004	0.013 (1.40)	0.003 (0.38)	0.008 (0.89)	0.013 (1.43)⁎
Percent change in population proportion of ≥60 years	31.88 (3.97)	0.074	0.010 (1.14)	0.019 (2.22)	0.021 (2.34)	0.026 (2.92)⁎

⁎ *P*<0.05 stands for statistical significance of the corresponding *β* estimates. *R*^2^ stands for the variance explained by each predictor; *β* stands for the coefficient in the linear regression model; 1991−2023 stands for the model includes all data from 1991−2023. DALY. disability-adjusted life year; SE. Standard error

Stratified analyses were conducted to examine the associations between changes in the total fertility rate of women aged 15−49 years and changes in DALY rates of women’s cancers by cancer type and age group across G20 and its 98 locations **(**Additional file 1**:** Tables S8 and S9**)**. Changes in total fertility rate were significantly associated with changes in DALY rates across four cancer types and three age groups from 1991 to 2023 (all *P*-values<0.001). A consistent inverse association was observed between changes in total fertility rate and changes in DALY rates for breast, cervical, uterine, and ovarian cancers from 1991 to 2023 (all *P*-values<0.001) **(**Additional file 1**:** Table S8**)**. Notably, changes in total fertility rate were more strongly associated with changes in DALY rates for breast and ovarian cancers among women aged 15−49 years across multiple time points (the majority of *P*-values<0.05) **(**Additional file 1**:** Table S9**)**.

## Discussion

Based on GBD 2023 estimates, our study presents a comprehensive assessment of the burden of women’s cancers in the G20. In 2023, the incidence, prevalence, mortality, and DALYs from the 4 major women’s cancers reached 3.29, 26.71, 1.16, and 36.58 million, respectively. The QCI was 75.13, and the 5-year relative survival was 65.74% for women’s cancer. There was a significant increase in incidence, prevalence, mortality, and DALYs compared to 1990, particularly in the African Union. Decomposition analysis revealed that population growth was the main driver of the increases in incidence, prevalence, mortality, and DALYs for women’s cancers, followed by population aging. These demographic changes underscore the need for healthcare systems to prepare for an increasing demand for cancer services. The age-standardized incidence rate, QCI, and 5-year relative survival increased, while the age-standardized mortality and DALY rates decreased. Notably, dominance analyses highlighted the impact of changes in breast and cervical cancer prevalence, together with total fertility rates among women aged 15−49 years as key predictors of DALY rate changes for women’s cancer in G20. Moreover, a sustained and significant influence of total fertility rates was seen over time. These findings suggest the need to expand targeted prevention, early detection, referral, treatment, and follow-up strategies, particularly focusing on reproductive health and fertility for women of reproductive age and improved cancer care for older women.

Population growth and population aging were the first and second contributors, respectively, to the absolute number increase in the burden of women’s cancer in the G20. However, epidemiological changes negatively impacted mortality and DALYs. This finding aligned with the previous studies reporting that the increase in DALYs from non-communicable diseases was due to the aging and growing global population [12], [19]. This trend is particularly concerning in the context of predictable population aging, as it suggests that the demand for cancer healthcare services will continue to rise, further straining already overburdened systems [33]. Among the 4 types, breast cancer continues to present the highest burden, with 18.79 million DALYs, followed by cervical cancer with 10.55 million DALYs, and ovarian cancer with 4.95 million DALYs. Uterine cancer ranks fourth despite having higher incidence and prevalence rates than ovarian cancer, likely due to ovarian cancer’s late diagnosis and more aggressive nature [9], [34] as seen with its lower QCI and 5-year relative survival.

The trend of increased age-standardized incidence rate but decreased age-standardized DALY rate in G20 from 1990 to 2023 is consistent with findings from previous studies on these cancer types [8], [10], [15]. The increases in age-standardized incidence rates of women’s cancer can be attributed to demographic shifts, unhealthy lifestyles, reproductive and hormonal changes, exposure to environmental, occupational, or genetic risk, and increasing national screening practices that report more cases [2], [3], [10], [15], [34], [35]. The decreases in age-standardized DALY rates suggest progress in enhancing the quality of care and health services with increased survival [8], [9]. In the stratified analysis, substantial decreases in age-standardized DALY rates were observed in breast and cervical cancers in women aged 50−69 years. This trend could be attributed to advances in cancer prevention policies, cancer treatment, quality of cancer care, and a better understanding of cancer epidemiology, leading to more effective interventions. Collectively, these factors would contribute to a long-term reduction in women’s cancer burden, reflecting a positive shift in health outcomes.

Substantial geographical variations were observed, with higher age-standardized DALY rates and lower QCI and 5-year relative survival in the African Union. These disparities are partly due to limited cancer registries, late diagnosis, limited treatment access [10], [15], and prevalent risk factors like limited human papillomavirus vaccination and higher human immunodeficiency virus (HIV) prevalence [15], [34]. From an interdisciplinary perspective, addressing these disparities requires a comprehensive approach that integrates demographic trends, healthcare access, health infrastructure development, targeted prevention and screening programs, and reproductive health policies. For instance, the African Union will require significant investments in health infrastructure to manage the expected increase in cancer cases due to population growth, the HIV epidemic, and suboptimal screening [36], [37].

Furthermore, the quality of care is crucial to addressing these disparities, including comprehensive early assessments, detection of asymptomatic and co-existing conditions, accurate diagnosis, timely and appropriate treatment, referral for hospital care and surgery when needed, and the ability for patients to follow up and adjust treatments as necessary [38], [39]. Saudi Arabia and South Africa showed the greatest increases in incidence, prevalence, and mortality, mainly driven by population growth, then epidemiological change, partly due to the prevalence of smoking, obesity, and environmental pollution [3], [15], [34], [40]. Improvements in reporting may also contribute. In contrast, the United Kingdom and European Union experienced decreases in DALYs, attributed to better healthcare infrastructure and higher rates of cancer screening [3], [15], [35], [38], [39]. That small-population countries like Eswatini, Equatorial Guinea, and Lesotho have extreme values should not be overinterpreted, since even a single case can distort their statistics, given the comparatively small populations of these nations. This underscores the importance of robust health systems and early intervention strategies of the high-income countries, which are essential to better quality care, significantly improved survival rates, and reduced disease burden. For lower-income countries, particularly in the African Union, significant investments in health infrastructure are necessary to improve test and treatment accessibility. This will promote healthy aging and manage the expected increase in cancer cases.

The burden of women’s cancers exhibited distinct age-related patterns and varying efficacy of control policies across age groups [10], [27], [41], [42]. Women of reproductive age (15−49 years) experienced excess DALYs ratios (0.93-fold and 2.29-fold compared to 50−69 years and ≥70 years) compared to incidence ratios (0.72-fold and 1.33-fold compared to 50−69 years and ≥70 years), with commensurate compromised health and reduced fertility [12], [18]. Previous studies mainly focused on fertility preservation in cancer patients [43], [44], but this study provides quantifiable empirical evidence on the decline in fertility and changes in women’s cancer burden at the population level. Changes in total fertility rates among women aged 15−49 years were consistently a dominant predictor of DALY rate changes. Lower fertility rates are associated with changes in hormonal profiles [45]. Reduced pregnancies and breastfeeding can lead to prolonged exposure to endogenous hormones, increasing the risk of conditions such as endometriosis and oligomenorrhea, which may elevate the risk of breast, ovarian, and uterine cancers [45], [46]. Conversely, higher parity may lower the risk of these cancers by modulating hormonal levels. These mechanisms align with our stratified analysis, which shows a negative association between fertility change and all 4 types of women’s cancer burden. This suggests that efforts to reduce the prevalence of breast and cervical cancers in women of reproductive age could substantially decrease the overall burden of women’s cancers. Furthermore, the dominance of the total fertility rate (7.35% in 2000, 12.05% in 2010, and 8.91% in 2023) indicates a significant influence of fertility patterns on women’s health outcomes. This finding provides a novel perspective on the bidirectional relationship between fertility decline and women’s cancer burden, underscoring the need for targeted interventions among women of reproductive age. These include subsidized screening and treatment services, improved fertility services, and other efforts to reduce women’s cancer burden.

These findings highlight that a better understanding of epidemiological changes is essential for informed healthcare planning and resource allocation. Incorporating socioeconomics and public health policy insights can enhance intervention strategies, ensuring resource optimization for regions with the highest burden of women's cancers. As emphasized in the WHO’s “Reproductive Health Strategy”, integrating reproductive health policies into national health plans is crucial for ensuring accessible and comprehensive services. Collaboration between governments and international organizations is essential to implement targeted policies, improve healthcare access, and reduce disparities, ultimately mitigating the burden of women’s cancers.

A major strength of this study is the use of dominance analysis to quantify the contribution of the key factors to the changes in DALY rates of women’s cancers. Dominance analysis identified several key predictors that may lower the women’s cancer burden, emphasizing the importance of prevention, early detection, and treatment, especially for women of reproductive age for the first time. However, this study has limitations. First, it relies on GBD 2023 estimates, which use modeling rather than direct observation and are subject to inherent data quality biases [47]. Second, the COVID-19 pandemic significantly disrupted cancer registries, screening, and treatment services, particularly in low-income regions. While our 1990−2023 trend analysis remains robust, the pandemic-induced “sharp decrease” in diagnoses likely reflects a significant backlog rather than a true decline in incidence[48]. Consequently, we anticipate a substantial increase in excess mortality across all malignancies, including women’s cancers, which are currently under-represented or inadequately captured in existing GBD models due to the projected backlog in diagnosis and delayed treatment. Nonetheless, we believe this study provides crucial insights into the evolving landscape of women’s cancer burden and its driving factors.

## Conclusions

In conclusion, the incidence, prevalence, mortality, and DALYs of the 4 major women’s cancers have increased significantly since 1990, with substantial geographical variations across G20 countries despite improvements in quality of care and survival. Strengthening breast and cervical cancer control, together with targeted support for reproductive health and fertility among women aged 15−49 years, should be prioritized as high-impact strategies to reduce national DALY burdens. G20 health systems should adopt differentiated, data-driven approaches that align cancer control with population aging, fertility transitions, and country-specific risk profiles to achieve sustainable and equitable reductions in women’s cancer burden.

## Abbreviations

AAPC: Average annual percent change

ASR: Age-standardized rate

DALY: Disability-adjusted life year

G20: The group of twenty

HIV: Human immunodeficiency virus

ICD: International Classification of Diseases

MIR: Mortality-to-incidence ratio

QCI: Quality of care index

SDI: Socio-demographic index

UI: Uncertainty interval

YLDs: Years lived with disability

YLLs: Years of life lost

## Ethics approval and consent to participate

Not applicable.

## Funding

This work was supported by the High-Level Public Health Specialized Talents Project of the Beijing Municipal Health Commission (2024-3-028), and in part by the U.S. National Institutes of Health (P30CA016359).

## Data Availability

The datasets generated and/or analyzed during the current study are not publicly available but are available from the corresponding author with valid rationales for their use.
