# Supplementary material for: Burden of women’s cancers in the group of twenty (G20) from 1990 to 2023: epidemiological trends and impact from fertility, quality of care, and survival
Source: Mil Med Res. 2026 Apr 27;13(1):100026. doi: 10.1016/j.mmr.2026.100026 (PMC13138205; doi:10.1016/j.mmr.2026.100026)
Supplement: Supplementary file 1 — Supplementary material [file mmc1.pdf]

Table S1 Prevalence, YLL, and YLD of women's cancers by type and age in 2023 and trends from 1990 to 2023 in G20

Characteristics	Prevalence			YLL			YLD		
	**Number (million, 95% UI)**	**ASR (/100,000, 95% UI)**	**AAPC (95% CI)**	**Number (million, 95% UI)**	**ASR (/100,000, 95% UI)**	**AAPC (95% CI)**	**Number (million, 95% UI)**	**ASR (/100,000, 95% UI)**	**AAPC (95% CI)**
Women's cancer	26.71 (21.99–32.40)	706.16 (555.75–890.02)	0.23 (0.17–0.30)	34.61 (26.79–43.91)	942.56 (685.88–1264.87)	–0.31 (–0.40 to –0.22)	1.97 (1.32–2.83)	52.24 (33.45–77.56)	0.19 (0.11–0.27)
Type									
Breast cancer	18.39 (15.83–21.21)	477.47 (393.37–572.41)	0.13 (0.06–0.20)	17.49 (14.10–21.12)	473.70 (357.48–606.62)	–0.17 (–0.27 to –0.07)	1.30 (0.89–1.83)	34.08 (22.49–49.37)	0.18 (0.09–0.28)
Cervical cancer	3.96 (2.70–5.58)	115.19 (75.55–167.69)	0.33 (0.15–0.52)	10.24 (7.29–14.15)	288.16 (194.41–418.15)	–0.37 (–0.50 to –0.24)	0.31 (0.19–0.49)	8.87 (5.14–14.37)	0.13 (–0.03 to 0.28)
Uterine cancer	3.28 (2.63–4.26)	83.87 (64.79–110.97)	0.74 (0.61–0.88)	2.07 (1.61–2.71)	52.76 (39.20–71.57)	–0.90 (–0.99 to –0.80)	0.22 (0.15–0.32)	5.63 (3.55–8.44)	0.56 (0.43–0.69)
Ovarian cancer	1.08 (0.84–1.36)	29.64 (22.03–38.95)	0.02 (–0.06 to 0.10)	4.81 (3.79–5.93)	127.95 (94.79–168.53)	–0.35 (–0.45 to –0.25)	0.14 (0.09–0.19)	3.65 (2.26–5.38)	–0.19 (–0.28 to –0.09)
Age group (years)									
15–49	7.92 (5.75–10.71)	495.56 (358.94–671.69)	0.55 (0.50–0.60)	13.93 (9.84–19.26)	867.34 (611.59–1200.91)	0.20 (0.08–0.32)	0.60 (0.37–0.94)	37.74 (23.16–58.72)	0.51 (0.46–0.56)
50–69	12.05 (9.76–14.84)	1931.13 (1563.95–2379.71)	0.16 (0.02–0.31)	14.80 (11.00–19.57)	2382.36 (1770.06–3152.11)	–0.68 (–0.81 to –0.54)	0.88 (0.57–1.29)	141.59 (91.34–207.70)	0.08 (–0.08 to 0.23)
≥ 70	6.74 (5.66–7.89)	2652.17 (2225.91–3101.91)	–0.10 (–0.14 to –0.06)	5.88 (4.44–7.42)	2308.74 (1744.23–2914.40)	–0.67 (–0.88 to –0.46)	0.48 (0.33–0.68)	189.99 (128.18–266.73)	–0.07 (–0.13 to –0.00)

The AAPC reflects trends in ASR from 1990 to 2023, while all other data pertain to 2023. Women's cancer refers to a combination of breast cancer, cervical cancer, uterine cancer, and ovarian cancer; AAPC. Average annual percent change; ASR. Age-standardized rate

Table S2 Incidence, mortality, DALY, QCI, and 5-year relative survival of women's cancers by location in 2023 and trends from 1990 to 2023 in G20

**Parameters**	**Incidence**			**Mortality**			**DALY**			**QCI**		**5-year relative survival**	
	**Number (million, 95% UI)**	**ASR (/100,000, 95% UI)**	**AAPC (95% CI)**	**Number (million, 95% UI)**	**ASR (/100,000, 95% UI)**	**AAPC (95% CI)**	**Number (million, 95% UI)**	**ASR (/100,000, 95% UI)**	**AAPC (95% CI)**	**Estimate (95% CI)**	**AAPC (95% CI)**	**Rate (%, 95% CI)**	**AAPC (95% CI)**
Argentina	0.03<br>(0.03–0.04)	103.74<br>(88.36–120.35)	0.44<br>(0.12–0.75)	0.01<br>(0.01–0.02)	41.36<br>(36.40–46.82)	–0.62<br>(–0.80 to –0.45)	0.38<br>(0.34–0.43)	1257.03<br>(1110.98–1406.86)	–0.45<br>(–0.76 to –0.13)	72.10<br>(69.29–74.90)	0.75<br>(0.65–0.84)	60.13<br>(58.98–61.28)	0.83<br>(0.73–0.93)
Australia	0.02<br>(0.02–0.03)	108.93<br>(94.94–123.92)	–0.04<br>(–0.19 to 0.10)	0.01<br>(0.01–0.01)	23.27<br>(20.16–25.94)	–1.59<br>(–1.82 to –1.36)	0.14<br>(0.13–0.16)	652.94<br>(579.84–729.72)	–1.82<br>(–1.87 to –1.78)	96.30<br>(92.98–99.62)	0.47<br>(0.42–0.51)	78.64<br>(78.36–78.92)	0.59<br>(0.57–0.61)
Brazil	0.11<br>(0.10–0.12)	77.77<br>(68.79–88.27)	0.52<br>(0.36–0.68)	0.04<br>(0.04–0.05)	30.71<br>(28.08–33.06)	–0.49<br>(–0.61 to –0.36)	1.35<br>(1.26–1.44)	964.73<br>(901.27–1027.69)	–0.35<br>(–0.56 to –0.14)	63.49<br>(59.43–67.55)	0.97<br>(0.91–1.03)	60.51<br>(58.82–62.19)	0.84<br>(0.78–0.90)
Canada	0.04<br>(0.04–0.05)	131.30<br>(111.26–154.83)	–0.34<br>(–0.54 to –0.15)	0.01<br>(0.01–0.01)	28.35<br>(24.88–32.01)	–1.25<br>(–1.56 to –0.95)	0.27<br>(0.24–0.31)	808.32<br>(720.32–907.91)	–1.36<br>(–1.73 to –1.00)	93.61<br>(90.30–96.91)	0.25<br>(0.24–0.26)	78.41<br>(77.56–79.25)	0.30<br>(0.26–0.34)
China	0.58<br>(0.43–0.75)	54.00<br>(40.63–69.26)	0.01<br>(–0.44 to 0.46)	0.16<br>(0.12–0.20)	14.09<br>(10.89–17.69)	–1.72<br>(–2.10 to –1.34)	4.97<br>(3.83–6.22)	459.10<br>(357.64–571.38)	–1.84<br>(–2.22 to –1.45)	85.16<br>(83.83–86.49)	1.12<br>(1.04–1.19)	73.91<br>(73.28–74.53)	0.97<br>(0.93–1.01)
France	0.10<br>(0.08–0.11)	155.33<br>(131.91–182.62)	0.83<br>(0.55–1.11)	0.03<br>(0.02–0.03)	31.76<br>(27.47–36.35)	–0.88<br>(–1.20 to –0.57)	0.58<br>(0.51–0.67)	912.20<br>(795.93–1042.64)	–0.91<br>(–1.17 to –0.64)	95.51<br>(92.50–98.51)	0.46<br>(0.42–0.49)	79.56<br>(79.10–80.01)	0.68<br>(0.65–0.71)
Germany	0.11<br>(0.10–0.13)	136.40<br>(121.28–152.34)	0.27<br>(0.09–0.44)	0.04<br>(0.03–0.04)	33.66<br>(29.97–37.01)	–1.13<br>(–1.25 to –1.02)	0.79<br>(0.70–0.87)	954.38<br>(862.16–1053.36)	–1.18<br>(–1.30 to –1.06)	88.51<br>(85.64–91.38)	0.40<br>(0.35–0.45)	75.32<br>(75.11–75.52)	0.63<br>(0.58–0.68)
India	0.41<br>(0.30–0.56)	58.81<br>(42.43–80.60)	1.01<br>(0.48–1.55)	0.21<br>(0.15–0.29)	31.83<br>(22.70–43.39)	0.37<br>(–0.17 to 0.91)	6.94<br>(5.04–9.42)	987.72<br>(720.03–1340.72)	0.22<br>(–0.30 to 0.75)	44.15<br>(42.32–45.98)	2.15<br>(1.73–2.57)	45.88<br>(45.42–46.33)	0.95<br>(0.89–1.01)
Indonesia	0.11<br>(0.07–0.17)	69.18<br>(44.48–103.63)	2.19<br>(1.98–2.40)	0.05<br>(0.03–0.07)	31.93<br>(20.65–46.74)	1.48<br>(1.28–1.68)	1.93<br>(1.25–2.85)	1183.49<br>(766.63–1744.20)	1.66<br>(1.45–1.87)	55.84<br>(53.89–57.78)	0.71<br>(0.64–0.78)	53.85<br>(53.19–54.52)	0.77<br>(0.73–0.81)
Italy	0.08<br>(0.06–0.09)	128.31<br>(108.17–149.41)	0.38<br>(0.04–0.72)	0.02<br>(0.02–0.03)	27.94<br>(24.05–31.54)	–0.81<br>(–1.20 to –0.42)	0.50<br>(0.43–0.56)	804.93<br>(708.10–901.71)	–0.94<br>(–1.24 to –0.65)	95.91<br>(92.64–99.17)	0.28<br>(0.26–0.30)	78.23<br>(77.66–78.79)	0.43<br>(0.38–0.47)
Japan	0.12<br>(0.10–0.14)	101.09<br>(88.17–114.94)	2.00<br>(1.65–2.35)	0.03<br>(0.03–0.04)	20.40<br>(17.73–22.51)	0.56<br>(0.39–0.73)	0.81<br>(0.69–0.90)	668.14<br>(596.69–736.25)	0.57<br>(0.32–0.81)	95.06<br>(91.59–98.53)	0.30<br>(0.28–0.32)	79.82<br>(79.48–80.16)	0.50<br>(0.47–0.53)
Republic of Korea	0.03<br>(0.02–0.03)	58.12<br>(41.07–76.65)	1.59<br>(1.33–1.85)	0.01<br>(0.00–0.01)	12.85<br>(9.21–16.55)	–0.71<br>(–0.88 to –0.55)	0.19<br>(0.13–0.24)	425.90<br>(303.25–546.16)	–0.80<br>(–1.07 to –0.53)	91.08<br>(89.88–92.28)	0.87<br>(0.78–0.97)	77.89<br>(77.47–78.30)	1.18<br>(1.11–1.26)
Mexico	0.05<br>(0.04–0.05)	63.66<br>(55.83–72.32)	–0.38<br>(–0.52 to –0.24)	0.02<br>(0.02–0.02)	25.85<br>(23.69–28.04)	–1.33<br>(–1.49 to –1.17)	0.61<br>(0.56–0.66)	807.22<br>(738.53–873.21)	–1.25<br>(–1.39 to –1.10)	62.39<br>(58.08–66.70)	1.37<br>(1.29–1.45)	59.40<br>(57.57–61.24)	0.90<br>(0.84–0.96)
Russian Federation	0.14<br>(0.12–0.17)	105.72<br>(90.47–123.42)	0.75<br>(0.45–1.04)	0.04<br>(0.04–0.05)	30.25<br>(26.98–33.91)	–0.35<br>(–0.77 to 0.08)	1.23<br>(1.10–1.38)	938.63<br>(840.37–1055.59)	–0.44<br>(–0.75 to –0.12)	81.74<br>(78.23–85.25)	0.57<br>(0.43–0.70)	71.39<br>(70.22–72.57)	0.58<br>(0.49–0.68)
Saudi Arabia	0.01<br>(0.00–0.01)	65.85<br>(46.95–91.24)	2.99<br>(2.74–3.25)	0.00<br>(0.00–0.00)	25.69<br>(18.37–34.05)	1.56<br>(1.29–1.83)	0.06<br>(0.04–0.09)	705.03<br>(506.27–1000.05)	1.43<br>(1.16–1.70)	71.77<br>(70.58–72.95)	0.87<br>(0.77–0.97)	60.99<br>(60.09–61.90)	1.44<br>(1.36–1.51)
South Africa	0.03<br>(0.03–0.05)	101.49<br>(77.19–137.54)	2.31<br>(1.82–2.81)	0.02<br>(0.01–0.02)	55.08<br>(42.94–70.97)	1.83<br>(1.32–2.34)	0.59<br>(0.45–0.80)	1745.81<br>(1339.92–2360.31)	1.97<br>(1.46–2.48)	53.66<br>(52.01–55.32)	0.97<br>(0.39–1.56)	45.73<br>(43.72–47.75)	0.67<br>(0.47–0.86)
Türkiye	0.03<br>(0.03–0.04)	63.04<br>(49.12–78.70)	1.34<br>(0.76–1.92)	0.01<br>(0.01–0.01)	21.54<br>(16.92–26.49)	–0.31<br>(–0.90 to 0.28)	0.34<br>(0.28–0.41)	648.48<br>(522.57–779.66)	–0.35<br>(–0.93 to 0.23)	77.09<br>(75.25–78.93)	1.30<br>(1.23–1.36)	65.83<br>(65.44–66.22)	1.44<br>(1.35–1.52)

Parameters	Incidence			Mortality			DALY			QCI		5-year relative survival	
	Number (million, 95% UI)	ASR (/100,000, 95% UI)	AAPC (95% CI)	Number (million, 95% UI)	ASR (/100,000, 95% UI)	AAPC (95% CI)	Number (million, 95% UI)	ASR (/100,000, 95% UI)	AAPC (95% CI)	Estimate (95% CI)	AAPC (95% CI)	Rate (%, 95% CI)	AAPC (95% CI)
United Kingdom	0.08 (0.07–0.09)	139.34 (122.18–158.15)	–0.61 (–0.75 to –0.48)	0.03 (0.02–0.03)	34.34 (30.85–37.24)	–1.52 (–1.68 to –1.35)	0.57 (0.51–0.62)	936.13 (863.35–1013.19)	–1.79 (–1.90 to –1.67)	91.18 (87.26–95.10)	0.36 (0.31–0.40)	75.36 (74.51–76.21)	0.38 (0.34–0.42)
United States of America	0.41 (0.35–0.49)	149.98 (127.73–177.35)	–0.67 (–0.78 to –0.57)	0.09 (0.08–0.10)	28.59 (25.13–32.05)	–1.22 (–1.44 to –1.01)	2.29 (2.03–2.58)	845.82 (756.09–952.67)	–1.40 (–1.46 to –1.34)	96.58 (93.22–99.95)	0.18 (0.16–0.20)	80.93 (80.13–81.74)	0.15 (0.13–0.17)
European Union	0.53 (0.46–0.60)	122.83 (108.18–138.60)	0.24 (0.13–0.36)	0.17 (0.15–0.18)	31.05 (27.84–33.89)	–1.05 (–1.14 to –0.97)	3.80 (3.40–4.16)	876.99 (792.95–962.76)	–1.21 (–1.29 to –1.12)	90.34 (86.92–93.77)	0.46 (0.44–0.49)	74.72 (74.08–75.36)	0.60 (0.58–0.62)
African Union	0.58 (0.41–0.79)	112.42 (80.69–152.45)	1.72 (1.60–1.85)	0.26 (0.19–0.36)	56.72 (40.04–76.44)	1.21 (1.11–1.31)	10.68 (7.55–14.44)	2045.85 (1456.81–2752.03)	1.20 (1.08–1.31)	46.60 (45.28–47.92)	1.01 (0.86–1.16)	49.54 (48.97–50.12)	0.63 (0.61–0.65)

The AAPC reflects trends in ASR, QCI, and 5-year relative survival rate from 1990 to 2023, while all other data pertain to 2023. SDI. Sociodemographic index; AAPC. Average annual percent change; ASR. Age-standardized rate; DALY. Disability-adjusted life year; QCI. Quality of care index; UI. Uncertainty interval; CI. Confidence interval

Table S3 Prevalence, YLL, and YLD of women's cancers by location in 2023 and trends from 1990 to 2023 in G20

Parameters	Prevalence			YLL			YLD		
	Number (million, 95% UI)	ASR (/100,000, 95% UI)	AAPC (95% CI)	Number (million, 95% UI)	ASR (/100,000, 95% UI)	AAPC (95% CI)	Number (million, 95% UI)	ASR (/100,000, 95% UI)	AAPC (95% CI)
Argentina	0.26 (0.22–0.29)	838.43 (726.62–966.56)	0.68 (0.42–0.95)	0.36 (0.32–0.41)	1193.50 (1051.83–1346.94)	–0.49 (–0.81 to –0.18)	0.02 (0.01–0.03)	63.53 (44.82–89.54)	0.60 (0.34–0.87)
Australia	0.24 (0.21–0.27)	1073.77 (958.73–1205.82)	0.13 (0.00–0.25)	0.13 (0.11–0.14)	577.53 (513.62–639.69)	–1.96 (–2.28 to –1.65)	0.02 (0.01–0.02)	75.42 (51.52–105.28)	0.01 (–0.12 to 0.15)
Brazil	0.74 (0.65–0.84)	531.20 (467.66–604.79)	0.88 (0.75–1.02)	1.29 (1.21–1.37)	925.73 (868.90–984.12)	–0.38 (–0.59 to –0.17)	0.05 (0.04–0.07)	39.01 (27.58–53.83)	0.68 (0.55–0.82)
Canada	0.42 (0.36–0.47)	1226.75 (1064.65–1412.53)	–0.25 (–0.40 to –0.11)	0.24 (0.22–0.27)	721.69 (643.02–811.40)	–1.46 (–1.84 to –1.08)	0.03 (0.02–0.04)	86.63 (59.23–122.07)	–0.39 (–0.56 to –0.22)
China	5.01 (4.02–6.17)	463.10 (370.66–572.91)	0.51 (0.25–0.77)	4.61 (3.55–5.79)	425.08 (330.44–530.28)	–1.96 (–2.34 to –1.57)	0.37 (0.25–0.53)	34.02 (22.87–49.56)	0.34 (0.08–0.60)
France	0.94 (0.82–1.07)	1483.96 (1294.73–1699.89)	0.97 (0.75–1.18)	0.52 (0.45–0.59)	805.13 (702.20–918.22)	–1.08 (–1.35 to –0.82)	0.07 (0.05–0.09)	107.07 (72.37–150.61)	0.91 (0.66–1.16)
Germany	1.03 (0.94–1.13)	1192.96 (1082.34–1312.59)	0.28 (0.12–0.43)	0.71 (0.64–0.78)	867.45 (784.95–952.73)	–1.29 (–1.40 to –1.17)	0.07 (0.05–0.10)	86.93 (60.02–117.65)	0.20 (0.03–0.36)
India	2.45 (1.84–3.27)	345.74 (260.41–459.73)	1.46 (1.16–1.76)	6.74 (4.88–9.18)	958.92 (697.75–1307.50)	0.19 (–0.34 to 0.73)	0.20 (0.13–0.31)	28.81 (18.53–43.50)	1.28 (0.92–1.64)
Indonesia	0.79 (0.53–1.13)	487.83 (331.15–690.84)	2.21 (1.75–2.67)	1.87 (1.21–2.75)	1145.08 (740.81–1687.25)	1.64 (1.43–1.85)	0.06 (0.04–0.10)	38.41 (22.45–60.63)	2.13 (1.64–2.61)
Italy	0.78 (0.68–0.89)	1267.22 (1107.62–1456.28)	0.40 (0.15–0.66)	0.44 (0.38–0.50)	715.72 (631.00–803.65)	–1.07 (–1.35 to –0.78)	0.05 (0.04–0.08)	89.21 (61.64–126.24)	0.33 (0.05–0.61)
Japan	1.24 (1.08–1.40)	972.02 (866.68–1089.10)	1.94 (1.73–2.16)	0.72 (0.62–0.80)	597.85 (536.59–656.58)	0.43 (0.19–0.67)	0.09 (0.06–0.12)	70.29 (47.02–98.63)	2.01 (1.74–2.27)
Republic of Korea	0.24 (0.18–0.29)	529.98 (397.41–664.96)	1.66 (1.46–1.86)	0.17 (0.12–0.22)	386.81 (274.44–497.85)	–0.98 (–1.25 to –0.70)	0.02 (0.01–0.03)	39.08 (24.88–58.52)	1.83 (1.58–2.08)
Mexico	0.33 (0.28–0.38)	431.34 (368.32–495.60)	0.21 (0.01–0.41)	0.59 (0.54–0.64)	775.01 (711.43–837.48)	–1.29 (–1.44 to –1.15)	0.02 (0.02–0.03)	32.21 (22.25–43.78)	–0.01 (–0.17 to 0.14)
Russian Federation	1.17 (1.02–1.35)	871.20 (748.72–1009.66)	0.93 (0.77–1.08)	1.15 (1.03–1.28)	875.43 (786.41–981.49)	–0.51 (–0.83 to –0.20)	0.09 (0.06–0.12)	63.20 (44.41–86.79)	0.86 (0.64–1.08)
Saudi Arabia	0.04 (0.03–0.06)	505.45 (399.70–667.47)	2.88 (2.63–3.12)	0.06 (0.04–0.08)	667.66 (478.28–950.89)	1.37 (1.09–1.64)	0.00 (0.00–0.01)	37.36 (24.26–57.33)	2.81 (2.46–3.17)
South Africa	0.25 (0.19–0.34)	728.58 (570.04–981.72)	2.88 (2.23–3.54)	0.57 (0.43–0.78)	1686.70 (1287.99–2287.04)	1.95 (1.44–2.46)	0.02 (0.01–0.03)	59.11 (38.76–85.45)	2.82 (2.17–3.47)
Türkiye	0.27 (0.22–0.33)	512.29 (408.63–613.58)	1.71 (1.31–2.10)	0.32 (0.26–0.39)	610.19 (489.25–739.38)	–0.44 (–1.02 to 0.15)	0.02 (0.01–0.03)	38.29 (25.68–54.83)	1.56 (1.05–2.06)

Parameters	Prevalence			YLL			YLD		
	Number (million, 95% UI)	ASR (/100,000, 95% UI)	AAPC (95% CI)	Number (million, 95% UI)	ASR (/100,000, 95% UI)	AAPC (95% CI)	Number (million, 95% UI)	ASR (/100,000, 95% UI)	AAPC (95% CI)
United Kingdom	0.77 (0.68–0.86)	1285.09 (1133.09–1441.67)	–0.43 (–0.54 to –0.32)	0.51 (0.47–0.55)	843.14 (780.52–900.75)	–1.90 (–2.03 to –1.77)	0.06 (0.04–0.08)	92.99 (64.71–130.62)	–0.53 (–0.65 to –0.40)
United States of America	4.05 (3.55–4.63)	1445.12 (1259.50–1662.47)	–0.55 (–0.65 to –0.44)	2.02 (1.79–2.25)	745.84 (669.27–831.06)	–1.47 (–1.53 to –1.40)	0.28 (0.19–0.38)	99.98 (69.04–137.83)	–0.67 (–0.77 to –0.57)
European Union	4.94 (4.44–5.48)	1131.93 (1013.67–1261.71)	0.34 (0.30–0.39)	3.45 (3.11–3.76)	795.51 (720.31–867.54)	–1.33 (–1.40 to –1.26)	0.35 (0.24–0.48)	81.48 (56.19–112.65)	0.30 (0.18–0.42)
African Union	3.76 (2.67–5.17)	709.52 (514.81–961.81)	1.96 (1.81–2.12)	10.39 (7.31–14.02)	1988.65 (1410.46–2678.24)	1.18 (1.06–1.30)	0.30 (0.18–0.46)	57.20 (35.76–87.54)	1.87 (1.70–2.04)

The AAPC reflects trends in ASR from 1990 to 2023, while all other data pertain to 2023. AAPC. Average annual percent change; ASR. Age-standardized rate; YLL. Year of life lost; YLD. Year lived with disability; UI. Uncertainty interval; CI. Confidence interval

Table S4 Temporal trends in age-standardized incidence, prevalence, mortality, DALY rates, QCI, and 5-year relative survival of women's cancers from 1990 to 2023 in G20 and its 98 locations

Parameters	AAPC (95% CI)					
	Incidence	Prevalence	Mortality	DALY	QCI	5-year relative survival
G20	0.21 (0.07–0.34)	0.23 (0.17–0.30)	–0.45 (–0.57 to –0.34)	–0.28 (–0.38 to –0.19)	0.27 (0.22–0.33)	0.41 (0.37–0.44)
Argentina	0.44 (0.12–0.75)	0.68 (0.42–0.95)	–0.62 (–0.80 to –0.45)	–0.45 (–0.76 to –0.13)	0.75 (0.65–0.84)	0.83 (0.73–0.93)
Australia	–0.04 (–0.19 to 0.10)	0.13 (0.00–0.25)	–1.59 (–1.82 to –1.36)	–1.82 (–1.87 to –1.78)	0.47 (0.42–0.51)	0.59 (0.57–0.61)
Brazil	0.52 (0.36–0.68)	0.88 (0.75–1.02)	–0.49 (–0.61 to –0.36)	–0.35 (–0.56 to –0.14)	0.97 (0.91–1.03)	0.84 (0.78–0.90)
Canada	–0.34 (–0.54 to –0.15)	–0.25 (–0.40 to –0.11)	–1.25 (–1.56 to –0.95)	–1.36 (–1.73 to –1.00)	0.25 (0.24–0.26)	0.30 (0.26–0.34)
China	0.01 (–0.44 to 0.46)	0.51 (0.25–0.77)	–1.72 (–2.10 to –1.34)	–1.84 (–2.22 to –1.45)	1.12 (1.04–1.19)	0.97 (0.93–1.01)
France	0.83 (0.55–1.11)	0.97 (0.75–1.18)	–0.88 (–1.20 to –0.57)	–0.91 (–1.17 to –0.64)	0.46 (0.42–0.49)	0.68 (0.65–0.71)
Germany	0.27 (0.09–0.44)	0.28 (0.12–0.43)	–1.13 (–1.25 to –1.02)	–1.18 (–1.30 to –1.06)	0.40 (0.35–0.45)	0.63 (0.58–0.68)
India	1.01 (0.48–1.55)	1.46 (1.16–1.76)	0.37 (–0.17 to 0.91)	0.22 (–0.30 to 0.75)	2.15 (1.73–2.57)	0.95 (0.89–1.01)
Indonesia	2.19 (1.98–2.40)	2.21 (1.75–2.67)	1.48 (1.28–1.68)	1.66 (1.45–1.87)	0.71 (0.64–0.78)	0.77 (0.73–0.81)
Italy	0.38 (0.04–0.72)	0.40 (0.15–0.66)	–0.81 (–1.20 to –0.42)	–0.94 (–1.24 to –0.65)	0.28 (0.26–0.30)	0.43 (0.38–0.47)
Japan	2.00 (1.65–2.35)	1.94 (1.73–2.16)	0.56 (0.39–0.73)	0.57 (0.32–0.81)	0.30 (0.28–0.32)	0.50 (0.47–0.53)
Republic of Korea	1.59 (1.33–1.85)	1.66 (1.46–1.86)	–0.71 (–0.88 to –0.55)	–0.80 (–1.07 to –0.53)	0.87 (0.78–0.97)	1.18 (1.11–1.26)
Mexico	–0.38 (–0.52 to –0.24)	0.21 (0.01–0.41)	–1.33 (–1.49 to –1.17)	–1.25 (–1.39 to –1.10)	1.37 (1.29–1.45)	0.90 (0.84–0.96)
Russian Federation	0.75 (0.45–1.04)	0.93 (0.77–1.08)	–0.35 (–0.77 to 0.08)	–0.44 (–0.75 to –0.12)	0.57 (0.43–0.70)	0.58 (0.49–0.68)
Saudi Arabia	2.99 (2.74–3.25)	2.88 (2.63–3.12)	1.56 (1.29–1.83)	1.43 (1.16–1.70)	0.87 (0.77–0.97)	1.44 (1.36–1.51)
South Africa	2.31 (1.82–2.81)	2.88 (2.23–3.54)	1.83 (1.32–2.34)	1.97 (1.46–2.48)	0.97 (0.39–1.56)	0.67 (0.47–0.86)
Türkiye	1.34 (0.76–1.92)	1.71 (1.31–2.10)	–0.31 (–0.90 to 0.28)	–0.35 (–0.93 to 0.23)	1.30 (1.23–1.36)	1.44 (1.35–1.52)
United Kingdom	–0.61 (–0.75 to –0.48)	–0.43 (–0.54 to –0.32)	–1.52 (–1.68 to –1.35)	–1.79 (–1.90 to –1.67)	0.36 (0.31–0.40)	0.38 (0.34–0.42)
United States of America	–0.67 (–0.78 to –0.57)	–0.55 (–0.65 to –0.44)	–1.22 (–1.44 to –1.01)	–1.40 (–1.46 to –1.34)	0.18 (0.16–0.20)	0.15 (0.13–0.17)
European Union	0.24 (0.13–0.36)	0.34 (0.30–0.39)	–1.05 (–1.14 to –0.97)	–1.21 (–1.29 to –1.12)	0.46 (0.44–0.49)	0.60 (0.58–0.62)
Austria	–0.29 (–0.51 to –0.07)	–0.13 (–0.18 to –0.07)	–1.54 (–1.75 to –1.32)	–1.82 (–2.01 to –1.63)	0.47 (0.44–0.51)	0.58 (0.55–0.61)
Belgium	–0.63 (–0.84 to –0.42)	–0.41 (–0.66 to –0.16)	–1.61 (–1.88 to –1.34)	–1.84 (–2.04 to –1.63)	0.42 (0.39–0.45)	0.44 (0.40–0.47)

Parameters	AAPC (95% CI)					
	Incidence	Prevalence	Mortality	DALY	QCI	5-year relative survival
Bulgaria	0.41 (0.02–0.80)	0.37 (0.11–0.63)	–0.15 (–0.46 to 0.17)	–0.37 (–0.77 to 0.02)	0.29 (0.13–0.45)	0.31 (0.16–0.45)
Croatia	0.25 (–0.01 to 0.50)	0.60 (0.37–0.82)	–1.01 (–1.30 to –0.71)	–0.98 (–1.24 to –0.72)	0.70 (0.62–0.77)	0.76 (0.68–0.85)
Cyprus	1.43 (1.06–1.81)	1.47 (1.22–1.71)	–0.25 (–0.57 to 0.06)	–0.42 (–0.77 to –0.07)	0.60 (0.52–0.67)	1.00 (0.94–1.06)
Czechia	–0.27 (–0.59 to 0.06)	0.10 (–0.20 to 0.40)	–1.52 (–1.80 to –1.24)	–1.65 (–1.92 to –1.37)	0.74 (0.67–0.82)	0.75 (0.67–0.83)
Denmark	–0.95 (–1.38 to –0.51)	–0.62 (–0.97 to –0.28)	–2.15 (–2.32 to –1.99)	–2.56 (–2.71 to –2.40)	0.63 (0.59–0.66)	0.64 (0.59–0.69)
Estonia	–0.32 (–0.78 to 0.13)	0.14 (–0.30 to 0.58)	–1.47 (–1.86 to –1.08)	–1.81 (–2.02 to –1.60)	0.76 (0.67–0.85)	0.69 (0.63–0.76)
Finland	0.45 (0.25–0.65)	0.62 (0.43–0.82)	–1.03 (–1.25 to –0.82)	–1.22 (–1.47 to –0.97)	0.40 (0.36–0.44)	0.56 (0.51–0.60)
Greece	–0.09 (–0.32 to 0.13)	–0.09 (–0.21 to 0.03)	–0.71 (–0.97 to –0.46)	–0.84 (–1.11 to –0.58)	0.18 (0.13–0.22)	0.23 (0.18–0.28)
Hungary	0.08 (–0.15 to 0.30)	0.45 (0.30–0.60)	–0.96 (–1.06 to –0.87)	–1.15 (–1.26 to –1.04)	0.75 (0.70–0.80)	0.73 (0.66–0.81)
Ireland	0.11 (–0.44 to 0.66)	0.26 (–0.02 to 0.53)	–1.35 (–1.74 to –0.96)	–1.60 (–1.85 to –1.36)	0.54 (0.50–0.57)	0.64 (0.59–0.69)
Latvia	–0.15 (–0.60 to 0.31)	0.13 (–0.31 to 0.56)	–0.85 (–1.24 to –0.46)	–1.04 (–1.42 to –0.66)	0.51 (0.36–0.65)	0.45 (0.26–0.63)
Lithuania	–0.28 (–0.47 to –0.10)	–0.07 (–0.27 to 0.12)	–1.05 (–1.45 to –0.65)	–1.23 (–1.72 to –0.74)	0.45 (0.38–0.52)	0.37 (0.27–0.46)
Luxembourg	–0.61 (–0.99 to –0.24)	–0.43 (–0.70 to –0.15)	–2.03 (–2.37 to –1.68)	–2.36 (–2.72 to –2.01)	0.58 (0.49–0.67)	0.64 (0.54–0.73)
Malta	–0.27 (–0.54 to 0.01)	–0.11 (–0.18 to –0.05)	–1.38 (–1.63 to –1.12)	–1.48 (–1.75 to –1.21)	0.46 (0.39–0.53)	0.62 (0.58–0.66)
Netherlands	–0.28 (–0.38 to –0.17)	0.02 (–0.07 to 0.10)	–1.53 (–1.66 to –1.41)	–1.74 (–1.94 to –1.54)	0.51 (0.47–0.54)	0.58 (0.54–0.62)
Poland	0.25 (0.11–0.39)	0.62 (0.40–0.83)	–0.64 (–0.82 to –0.47)	–1.02 (–1.18 to –0.86)	0.91 (0.85–0.97)	0.75 (0.66–0.84)
Portugal	0.53 (0.02–1.04)	0.76 (0.50–1.03)	–1.30 (–1.52 to –1.07)	–1.37 (–1.75 to –0.98)	0.53 (0.39–0.67)	0.66 (0.63–0.70)
Romania	0.45 (0.26–0.64)	0.68 (0.55–0.80)	–0.19 (–0.39 to 0.02)	–0.58 (–0.75 to –0.42)	0.66 (0.57–0.75)	0.51 (0.39–0.62)
Slovakia	0.45 (0.27–0.64)	0.59 (0.49–0.69)	–0.39 (–0.56 to –0.23)	–0.64 (–0.73 to –0.56)	0.56 (0.53–0.59)	0.56 (0.52–0.59)
Slovenia	0.00 (–0.14 to 0.14)	0.39 (0.26–0.52)	–1.38 (–1.57 to –1.18)	–1.66 (–1.92 to –1.40)	0.73 (0.66–0.79)	0.75 (0.64–0.85)
Spain	–0.06 (–0.32 to 0.20)	0.08 (–0.13 to 0.28)	–1.42 (–1.54 to –1.30)	–1.54 (–1.76 to –1.32)	0.42 (0.39–0.45)	0.55 (0.51–0.59)
Sweden	–0.28 (–0.50 to –0.05)	–0.14 (–0.42 to 0.14)	–1.35 (–1.55 to –1.15)	–1.67 (–1.75 to –1.59)	0.35 (0.27–0.43)	0.43 (0.32–0.54)
African Union	1.72 (1.60–1.85)	1.96 (1.81–2.12)	1.21 (1.11–1.31)	1.20 (1.08–1.31)	1.01 (0.86–1.16)	0.63 (0.61–0.65)
Burundi	1.46 (1.25–1.67)	1.34 (1.03–1.65)	1.20 (0.92–1.47)	1.01 (0.79–1.23)	0.42 (0.13–0.71)	0.32 (0.23–0.41)
Cameroon	1.19 (0.89–1.48)	1.39 (1.07–1.72)	0.79 (0.43–1.14)	0.73 (0.37–1.09)	1.17 (0.88–1.47)	0.61 (0.42–0.79)

Parameters	AAPC (95% CI)					
	Incidence	Prevalence	Mortality	DALY	QCI	5-year relative survival
Central African Republic	1.26 (0.94–1.59)	1.05 (0.65–1.44)	1.09 (0.74–1.43)	1.11 (0.81–1.41)	–0.32 (–0.76 to 0.13)	0.38 (0.33–0.44)
Chad	0.72 (0.44–1.00)	0.82 (0.53–1.11)	0.52 (0.29–0.75)	0.61 (0.33–0.88)	0.62 (0.40–0.84)	0.33 (0.22–0.44)
Congo	1.74 (1.14–2.34)	1.07 (0.50–1.64)	1.62 (0.99–2.25)	1.28 (0.67–1.89)	–0.62 (–0.87 to –0.36)	0.24 (0.16–0.32)
Democratic Republic of the Congo	2.66 (2.45–2.88)	2.81 (2.62–3.00)	2.21 (2.01–2.41)	2.30 (2.08–2.51)	1.05 (0.90–1.21)	0.56 (0.49–0.63)
Equatorial Guinea	3.57 (2.92–4.23)	3.24 (2.26–4.23)	2.73 (2.19–3.28)	2.64 (2.02–3.26)	1.86 (1.02–2.71)	1.05 (0.91–1.19)
Gabon	2.30 (1.48–3.13)	2.38 (1.55–3.21)	1.77 (1.04–2.52)	1.71 (0.89–2.53)	0.90 (0.77–1.03)	0.60 (0.43–0.78)
São Tomé and Príncipe	1.82 (1.38–2.26)	1.86 (1.41–2.32)	1.30 (0.84–1.77)	1.21 (0.66–1.75)	1.20 (0.60–1.80)	0.60 (0.42–0.79)
Comoros	0.05 (–0.46 to 0.57)	0.24 (–0.13 to 0.61)	–0.18 (–0.38 to 0.03)	–0.29 (–0.77 to 0.20)	0.81 (0.52–1.10)	0.19 (0.08–0.30)
Djibouti	0.53 (0.11–0.95)	0.73 (0.29–1.18)	0.26 (–0.10 to 0.62)	0.16 (–0.24 to 0.57)	0.69 (0.55–0.83)	0.30 (0.19–0.40)
Eritrea	1.99 (1.83–2.15)	2.30 (2.06–2.55)	1.58 (1.36–1.79)	1.59 (1.37–1.82)	1.27 (1.04–1.49)	0.49 (0.47–0.52)
Ethiopia	2.89 (2.63–3.15)	2.93 (2.63–3.23)	2.39 (2.19–2.58)	2.18 (1.94–2.42)	1.28 (1.07–1.50)	0.62 (0.56–0.67)
Kenya	1.02 (0.85–1.19)	1.27 (1.07–1.47)	0.68 (0.48–0.89)	0.70 (0.53–0.87)	0.80 (0.63–0.97)	0.39 (0.30–0.47)
Madagascar	0.35 (–0.05 to 0.75)	0.52 (0.22–0.82)	0.22 (–0.12 to 0.56)	–0.04 (–0.42 to 0.35)	0.94 (0.76–1.11)	0.14 (0.05–0.23)
Mauritius	1.65 (1.01–2.28)	1.95 (1.42–2.49)	0.96 (0.35–1.58)	0.98 (0.36–1.60)	0.58 (0.47–0.69)	0.46 (0.34–0.59)
Rwanda	0.78 (0.27–1.29)	0.96 (0.39–1.53)	0.54 (0.20–0.88)	0.08 (–0.49 to 0.66)	1.38 (1.19–1.56)	0.21 (0.09–0.32)
Seychelles	1.02 (0.77–1.26)	1.49 (1.22–1.76)	0.28 (0.07–0.48)	0.38 (0.18–0.57)	1.06 (1.02–1.10)	0.78 (0.65–0.90)
Somalia	1.33 (1.09–1.57)	0.90 (0.70–1.09)	1.27 (1.05–1.49)	1.20 (1.00–1.41)	–0.52 (–0.82 to –0.22)	0.19 (0.12–0.26)
South Sudan	1.38 (0.97–1.79)	1.44 (1.02–1.86)	1.22 (0.82–1.61)	1.18 (0.75–1.62)	0.41 (0.26–0.56)	0.18 (0.13–0.23)
Sudan	2.02 (1.83–2.21)	2.19 (2.08–2.31)	0.84 (0.66–1.03)	1.08 (0.88–1.29)	1.16 (1.07–1.25)	1.41 (1.31–1.50)
United Republic of Tanzania	–0.49 (–0.83 to –0.14)	–0.14 (–0.43 to 0.16)	–0.56 (–0.80 to –0.33)	–0.87 (–1.22 to –0.52)	1.06 (0.71–1.43)	0.23 (0.12–0.34)
Uganda	2.10 (1.63–2.56)	1.49 (1.17–1.81)	1.56 (1.21–1.91)	1.70 (1.41–2.00)	–0.30 (–0.62 to 0.03)	0.63 (0.58–0.68)
Algeria	1.65 (1.23–2.08)	1.97 (1.81–2.14)	0.46 (0.33–0.59)	0.52 (0.38–0.65)	0.86 (0.83–0.89)	0.84 (0.81–0.87)
Egypt	3.58 (2.89–4.27)	3.39 (3.09–3.69)	2.51 (2.07–2.96)	2.21 (1.66–2.75)	0.79 (0.68–0.91)	1.22 (1.09–1.35)

Parameters	AAPC (95% CI)					
	**Incidence**	**Prevalence**	**Mortality**	**DALY**	**QCI**	**5-year relative survival**
Libya	1.40 (0.80–2.01)	1.62 (1.38–1.86)	0.66 (0.28–1.05)	0.65 (0.20–1.09)	0.57 (0.43–0.72)	0.58 (0.48–0.68)
Mauritania	2.91 (2.36–3.47)	3.21 (2.63–3.80)	2.15 (1.57–2.73)	2.25 (1.68–2.83)	1.46 (1.04–1.88)	1.06 (0.96–1.15)
Morocco	3.08 (2.95–3.21)	3.07 (2.89–3.26)	1.79 (1.69–1.89)	2.16 (2.05–2.28)	1.15 (0.97–1.33)	1.39 (1.32–1.46)
Tunisia	2.27 (2.05–2.49)	2.85 (2.22–3.48)	0.79 (0.57–1.01)	0.92 (0.68–1.16)	1.04 (0.87–1.22)	1.03 (0.99–1.07)
Angola	1.77 (1.20–2.34)	1.97 (1.28–2.66)	1.32 (0.79–1.86)	1.32 (0.73–1.93)	1.37 (1.06–1.68)	0.61 (0.53–0.68)
Botswana	2.47 (1.56–3.39)	1.73 (0.61–2.87)	1.74 (0.80–2.69)	1.80 (0.87–2.73)	0.19 (–2.38 to 2.83)	0.77 (0.59–0.95)
Eswatini	0.36 (–0.55 to 1.29)	0.98 (0.52–1.44)	0.31 (–0.78 to 1.41)	0.11 (–0.78 to 1.01)	1.19 (0.97–1.42)	0.05 (–0.10 to 0.19)
Lesotho	1.35 (0.18–2.54)	1.06 (0.29–1.84)	1.47 (0.66–2.28)	1.36 (0.13–2.60)	–0.01 (–2.20 to 2.23)	0.12 (–0.12 to 0.36)
Malawi	1.96 (1.09–2.83)	2.12 (1.68–2.57)	1.62 (0.70–2.55)	1.55 (0.67–2.44)	1.09 (0.28–1.90)	0.38 (0.25–0.50)
Mozambique	–0.93 (–1.11 to –0.75)	–0.47 (–1.17 to 0.23)	–0.58 (–0.79 to –0.38)	–1.20 (–1.40 to –1.00)	0.61 (0.32–0.91)	–0.41 (–0.45 to –0.36)
Namibia	1.71 (0.95–2.48)	2.16 (1.68–2.64)	1.14 (0.55–1.73)	1.02 (0.34–1.71)	1.18 (0.10–2.28)	0.45 (0.22–0.68)
Zambia	1.51 (1.14–1.87)	1.39 (1.01–1.77)	1.25 (0.94–1.57)	1.17 (0.81–1.52)	0.28 (–0.09 to 0.64)	0.35 (0.28–0.41)
Zimbabwe	1.75 (0.82–2.69)	1.26 (0.50–2.02)	1.50 (1.13–1.88)	1.99 (0.77–3.23)	–0.52 (–1.03 to 0.00)	0.37 (0.28–0.46)
Benin	1.41 (0.97–1.85)	1.82 (1.57–2.08)	0.75 (0.45–1.05)	0.99 (0.69–1.30)	1.23 (1.12–1.34)	0.97 (0.88–1.05)
Burkina Faso	0.95 (0.59–1.31)	1.13 (0.81–1.44)	0.58 (0.23–0.93)	0.59 (0.21–0.98)	1.19 (0.96–1.41)	0.66 (0.53–0.79)
Cabo Verde	1.11 (0.66–1.55)	0.69 (–0.62 to 2.01)	0.12 (–0.27 to 0.52)	0.37 (–0.05 to 0.79)	–0.01 (–0.16 to 0.14)	1.10 (0.93–1.27)
Côte d'Ivoire	2.17 (1.67–2.67)	2.60 (2.07–3.14)	1.59 (1.10–2.08)	1.87 (1.40–2.35)	1.42 (1.23–1.61)	0.99 (0.88–1.10)
Gambia	1.67 (1.12–2.22)	1.53 (1.08–1.98)	1.54 (1.12–1.96)	1.45 (1.01–1.89)	0.22 (–0.01 to 0.44)	0.34 (0.21–0.47)
Ghana	1.11 (0.77–1.46)	1.21 (0.83–1.58)	0.72 (0.43–1.02)	0.63 (0.29–0.97)	0.87 (0.69–1.04)	0.52 (0.45–0.59)
Guinea	1.12 (0.82–1.42)	1.45 (1.18–1.71)	0.69 (0.44–0.94)	0.86 (0.53–1.19)	1.48 (1.25–1.72)	0.63 (0.58–0.68)
Guinea-Bissau	1.95 (1.64–2.27)	2.07 (1.82–2.32)	1.74 (0.76–2.74)	1.69 (1.37–2.01)	0.80 (0.28–1.32)	0.54 (0.43–0.65)
Liberia	2.46 (2.00–2.92)	2.70 (2.31–3.10)	1.82 (1.32–2.33)	2.02 (1.56–2.48)	1.22 (1.06–1.38)	0.81 (0.69–0.93)

Parameters	AAPC (95% CI)					
	Incidence	Prevalence	Mortality	DALY	QCI	5-year relative survival
Mali	1.75 (1.23–2.27)	2.09 (1.71–2.47)	1.34 (0.73–1.95)	1.42 (0.85–1.98)	1.29 (1.13–1.45)	0.56 (0.49–0.62)
Niger	1.39 (1.08–1.70)	1.53 (1.24–1.82)	0.95 (0.69–1.22)	1.07 (0.77–1.37)	0.86 (0.75–0.98)	0.72 (0.66–0.77)
Nigeria	1.36 (1.10–1.61)	1.69 (1.46–1.92)	0.92 (0.60–1.23)	0.79 (0.36–1.22)	1.15 (1.01–1.28)	0.53 (0.47–0.58)
Senegal	2.08 (1.24–2.93)	2.20 (1.16–3.25)	1.86 (0.80–2.94)	1.60 (0.74–2.47)	0.84 (0.52–1.15)	0.48 (0.38–0.59)
Sierra Leone	1.10 (0.80–1.39)	1.33 (0.98–1.69)	0.76 (0.41–1.12)	0.76 (0.42–1.10)	1.02 (0.75–1.29)	0.43 (0.35–0.52)
Togo	1.59 (1.02–2.17)	1.75 (1.19–2.32)	1.15 (0.59–1.71)	1.19 (0.53–1.84)	0.96 (0.66–1.26)	0.67 (0.53–0.80)

AAPC. Average annual percent change; DALY. Disability-adjusted life year; QCI. Quality of care index; CI. Confidence interval

Table S5 Decomposition analysis of changes in incidence, prevalence, mortality and DALYs of women's cancers by location in 2023 in G20, compared with 1990 (%)

Parameters	Overall percent change	Population aging	Population growth	Epidemiological change
Incidence				
G20	117.95	34.08	74.67	9.20
Argentina	91.83	12.89	69.25	9.69
Australia	112.65	35.39	78.61	–1.35
Brazil	206.58	68.43	104.69	33.46
Canada	73.37	36.85	52.54	–16.01
China	114.37	68.50	48.71	–2.84
France	99.07	27.44	31.65	39.97
Germany	38.57	18.82	6.17	13.58
India	251.89	39.63	146.04	66.23
Indonesia	396.17	70.84	149.36	175.97
Italy	58.59	29.25	8.92	20.42
Japan	173.19	40.61	16.56	116.01
Republic of Korea	262.06	90.54	66.03	105.49
Mexico	153.02	63.01	110.50	–20.49
Russian Federation	61.13	18.24	9.08	33.81
Saudi Arabia	626.90	63.24	266.21	297.45
South Africa	448.06	59.68	185.49	202.90
Türkiye	299.63	77.19	131.11	91.33
United Kingdom	21.48	10.43	22.41	–11.36
United States of America	35.06	22.07	36.29	–23.31
European Union	53.24	25.00	14.55	13.70
African Union	367.98	15.56	217.12	135.29
Prevalence				
G20	121.48	35.76	75.36	10.35
Argentina	108.60	12.54	72.41	23.65
Australia	127.21	37.89	81.48	7.83
Brazil	225.50	65.30	108.03	52.16
Canada	80.55	38.92	53.67	–12.03
China	155.01	75.17	53.53	26.31
France	104.53	28.34	32.17	44.03
Germany	41.96	19.88	6.25	15.84
India	299.58	36.61	158.10	104.87
Indonesia	416.31	72.11	153.60	190.59
Italy	58.37	30.90	8.94	18.53
Japan	172.92	46.31	16.59	110.02
Republic of Korea	290.07	105.48	69.56	115.02
Mexico	190.43	62.41	117.97	10.05
Russian Federation	74.03	19.32	9.50	45.21

Parameters	Overall percent change	Population aging	Population growth	Epidemiological change
Saudi Arabia	618.44	64.96	263.66	289.81
South Africa	541.56	64.50	207.98	269.08
Türkiye	344.23	80.56	140.37	123.30
United Kingdom	27.10	10.45	22.91	–6.27
United States of America	43.09	23.69	37.30	–17.90
European Union	60.00	26.06	14.91	19.03
African Union	400.03	16.07	226.63	157.34
Mortality				
G20	85.25	38.64	68.94	–22.33
Argentina	53.50	19.83	62.05	–28.39
Australia	46.66	40.66	66.37	–60.37
Brazil	148.50	79.36	93.84	–24.69
Canada	47.86	47.34	49.05	–48.52
China	35.97	70.18	39.94	–74.15
France	27.23	29.72	25.01	–27.51
Germany	–1.89	23.51	5.21	–30.61
India	197.55	47.52	132.33	17.70
Indonesia	309.32	70.97	130.84	107.51
Italy	27.80	41.16	7.96	–21.32
Japan	120.94	72.45	14.33	34.16
Republic of Korea	110.40	96.01	48.30	–33.90
Mexico	99.53	68.46	99.89	–68.83
Russian Federation	16.27	19.62	7.61	–10.97
Saudi Arabia	320.29	35.75	184.43	100.11
South Africa	370.75	58.05	166.71	145.99
Türkiye	149.19	72.83	100.03	–23.67
United Kingdom	–5.72	13.08	20.02	–38.81
United States of America	18.01	24.91	34.32	–41.22
European Union	13.74	31.81	12.56	–30.62
African Union	297.33	14.63	196.18	86.51
DALYs				
G20	80.80	27.08	67.89	–14.17
Argentina	43.65	9.95	60.03	–26.33
Australia	23.38	26.70	61.41	–64.73
Brazil	126.40	55.91	88.74	–18.25
Canada	26.75	32.16	45.51	–50.92
China	16.31	50.61	36.70	–71.00
France	15.72	21.27	23.88	–29.42
Germany	–13.09	14.83	4.92	–32.85
India	166.93	31.01	124.92	11.00
Indonesia	308.86	61.55	131.06	116.26

Parameters	Overall percent change	Population aging	Population growth	Epidemiological change
Italy	4.38	24.38	7.20	–27.20
Japan	66.91	26.76	12.15	28.00
Republic of Korea	68.68	59.20	42.55	–33.08
Mexico	88.10	53.87	96.26	–62.03
Russian Federation	7.60	14.17	7.33	–13.90
Saudi Arabia	329.84	45.22	186.33	98.28
South Africa	387.57	55.35	171.03	161.18
Türkiye	120.95	52.57	93.74	–25.36
United Kingdom	–17.57	9.70	18.96	–46.23
United States of America	7.90	20.23	32.94	–45.27
European Union	–2.82	19.74	11.63	–34.19
African Union	291.10	14.29	194.24	82.56

DALY. Disability-adjusted life year

Table S6 Percent change of the indicators in G20 and its 98 locations, compared with 1990 [% (SE)]

**Predictors**	**2023 (*n*=99)**	**2010 (*n*=99)**	**2000 (*n*=99)**	**1991–2023 (*n*=3267)**
Percent change in DALY rate of women's cancer	46.75 (6.12)	8.80 (3.29)	1.09 (2.26)	10.80 (0.63)
Percent change in breast cancer prevalence rate of 15–49 years	116.69 (11.41)	35.25 (5.59)	12.57 (3.00)	36.19 (1.14)
Percent change in ovarian cancer prevalence rate of 15–49 years	82.35 (10.04)	25.16 (4.81)	12.21 (2.88)	26.38 (0.95)
Percent change in cervical cancer prevalence rate of 15–49 years	35.03 (7.01)	4.74 (3.82)	–4.11 (2.20)	6.23 (0.74)
Percent change in uterine cancer prevalence rate of 15–49 years	105.00 (8.37)	34.95 (5.71)	6.08 (2.93)	32.85 (1.05)
Percent change in total fertility rate of 15–49 years	–31.54 (1.69)	–17.30 (1.46)	–13.88 (1.13)	–16.96 (0.26)
Percent change in 5-year relative survival	22.58 (1.29)	12.60 (1.08)	4.79 (0.77)	10.38 (0.20)
Percent change in quality of care index	28.47 (2.06)	14.86 (1.97)	3.16 (1.66)	12.45 (0.36)
Percent change in total female population	78.66 (7.08)	41.58 (3.46)	18.11 (1.47)	36.24 (0.76)
Percent change in socio-demographic index	52.76 (4.79)	29.03 (2.26)	13.08 (0.95)	25.22 (0.52)
Percent change in population proportion of ≥60 years	31.88 (3.97)	9.09 (2.19)	5.31 (1.24)	10.92 (0.42)

DALY. Disability-adjusted life year; SE. Standard error

Table S7 Multivariable linear regression model of associations between the predictors and the change in DALY rates of women's cancers in G20 and its 98 locations

**Predictors**	**2023 (*n*=99)**		**2010 (*n*=99)**		**2000 (*n*=99)**		**1991–2023 (*n*=3267)**	
	β	***P*-value**	***β***	***P*-value**	***β***	***P*-value**	***β***	***P*-value**
Percent change in breast cancer prevalence rate of 15–49 years	0.336	<0.001	0.235	<0.001	0.250	<0.001	0.307	<0.001
Percent change in ovarian cancer prevalence rate of 15–49 years	0.124	0.020	0.125	0.030	–0.029	0.480	0.100	<0.001
Percent change in cervical cancer prevalence rate of 15–49 [illegible]	0.201	<0.001	0.361	<0.001	0.496	<0.001	0.282	<0.001
Percent change in uterine cancer prevalence rate of 15–49 years	–0.008	0.821	–0.072	0.059	0.039	0.445	–0.035	<0.001
Percent change in total fertility rate of 15–49 years	–0.456	0.004	–0.513	<0.001	–0.376	<0.001	–0.420	<0.001
Percent change in 5-year relative survival	–0.401	0.076	0.102	0.627	0.140	0.423	–0.071	0.029
Percent change in quality of care index	–0.680	<0.001	–0.543	<0.001	–0.614	<0.001	–0.575	<0.001
Percent change in total female population	0.057	0.223	0.042	0.516	0.189	0.017	0.039	<0.001
Percent change in socio-demographic index	0.004	0.943	–0.007	0.935	0.059	0.585	0.027	0.015
Percent change in population proportion of ≥60 years	0.074	0.295	0.073	0.389	0.052	0.493	0.079	<0.001

β stands for the coefficient in the linear regression model. DALY. Disability-adjusted life year

Table S8 Stratified linear regression model of associations between change in total fertility rate of 15–49 years and the change in DALY rates of women's cancers in G20 and its 98 locations

Group	2023 (*n*=99)			2010 (*n*=99)			2000 (*n*=99)			1991–2023 (*n*=3267)		
	R^2	*β*	*P*-	R^2	*β*	*P*–	R^2	*β*	*P*-	R^2	*β*	*P*-value
Type												
Breast cancer	0.222	–1.895	<0.001	0.188	–0.933	<0.001	0.114	–0.570	<0.001	0.231	–1.127	<0.001
Cervical cancer	0.087	–0.724	0.003	0.014	–0.228	0.238	0.022	–0.292	0.146	0.013	–0.207	<0.001
Uterine cancer	0.043	–0.466	0.039	<0.001	0.027	0.874	0.023	–0.168	0.134	0.035	–0.299	<0.001
Ovarian cancer	0.259	–2.397	<0.001	0.188	–0.969	<0.001	0.101	–0.613	0.001	0.242	–1.293	<0.001
Age group (years)												
15–49	0.258	–1.941	<0.001	0.127	–0.938	<0.001	0.132	–0.855	<0.001	0.172	–1.113	<0.001
50–69	0.214	–1.378	<0.001	0.147	–0.695	<0.001	0.081	–0.341	0.004	0.174	–0.742	<0.001
≥ 70	0.205	–1.141	<0.001	0.120	–0.624	<0.001	0.053	–0.305	0.022	0.172	–0.697	<0.001

R^2 stands for the variance explained by each predictor; *β* stands for the coefficient in the linear regression model. DALY. Disability-adjusted life year;

Table S9 Stratified linear regression model of associations between change in total fertility rate of 15–49 years and the change in DALY rates by age group and cancer type in G20 and its 98 locations

**Group**	**2023 (*n*=99)**			**2010 (*n*=99)**			**2000 (*n*=99)**			**1991–2023 (*n*=3267)**		
	R^2	β	***P*-value**	R^2	β	***P*-value**	R^2	β	***P*-value**	R^2	β	***P*-value**
15–49 years												
Breast cancer	0.261	–2.770	<0.001	0.155	–1.304	<0.001	0.116	–0.931	<0.001	0.221	–1.674	<0.001
Cervical cancer	0.102	–0.994	0.001	0.009	–0.241	0.356	0.052	–0.556	0.023	0.029	–0.419	<0.001
Uterine cancer	0.069	–0.888	0.009	0.002	0.130	0.661	0.040	–0.455	0.046	0.045	–0.587	<0.001
Ovarian cancer	0.287	–3.182	<0.001	0.181	–1.266	<0.001	0.176	–1.004	<0.001	0.249	–1.720	<0.001
50–69 years												
Breast cancer	0.228	–1.968	<0.001	0.168	–0.965	<0.001	0.090	–0.427	0.003	0.227	–1.154	<0.001
Cervical cancer	0.083	–0.722	0.004	0.017	–0.264	0.199	0.002	–0.069	0.707	0.014	–0.216	<0.001
Uterine cancer	0.047	–0.511	0.031	<0.001	–0.023	0.899	0.020	–0.154	0.165	0.038	–0.324	<0.001
Ovarian cancer	0.260	–2.480	<0.001	0.187	–1.042	<0.001	0.078	–0.539	0.005	0.244	–1.364	<0.001
≥ 70 years												
Breast cancer	0.197	–1.642	<0.001	0.119	–0.897	<0.001	0.090	–0.422	0.003	0.209	–1.120	<0.001
Cervical cancer	0.055	–1.095	0.020	0.013	–0.466	0.270	0.023	0.693	0.132	0.004	–0.276	<0.001
Uterine cancer	0.038	–0.395	0.054	<0.001	–0.024	0.894	0.010	–0.107	0.323	0.035	–0.296	<0.001
Ovarian cancer	0.160	–1.032	<0.001	0.090	–0.432	0.003	<0.001	0.021	0.904	0.075	–0.482	<0.001

R^2 stands for the variance explained by each predictor; β stands for the coefficient in the linear regression model. DALY. Disability-adjusted life year

Group	Location	Age-standardized incidence rate (1/100,000)					Age-standardized mortality rate (1/100,000)					Age-standardized DALY rate (1/100,000)				
		Women's cancer	Breast cancer	Cervical cancer	Uterine cancer	Ovarian cancer	Women's cancer	Breast cancer	Cervical cancer	Uterine cancer	Ovarian cancer	Women's cancer	Breast cancer	Cervical cancer	Uterine cancer	Ovarian cancer
G20	Argentina	103.74	58.99	28.99	8.02	7.74	41.36	23.87	10.00	2.32	5.17	1257.03	687.29	362.63	59.02	148.08
	Australia	108.93	74.45	9.66	17.14	7.67	23.27	14.37	2.03	2.22	4.64	652.94	413.83	67.61	57.83	113.67
	Brazil	77.77	44.05	19.79	8.17	5.75	30.71	16.57	7.78	2.55	3.82	964.73	516.07	271.07	65.31	112.29
	Canada	131.30	82.51	14.71	24.82	9.27	28.35	16.81	2.49	3.37	5.69	808.32	487.12	87.30	88.52	145.38
	China	54.00	31.62	12.06	6.66	3.66	14.09	6.76	4.19	1.11	2.04	459.10	228.95	133.06	34.64	62.45
	France	155.33	114.63	9.32	21.16	10.22	31.76	20.61	2.67	2.87	5.60	912.20	619.80	82.84	71.30	138.26
	Germany	136.40	100.41	9.53	16.54	9.92	33.66	22.15	2.91	2.44	6.17	954.38	640.68	95.18	61.39	157.13
	India	58.81	29.41	19.33	3.14	6.93	31.83	15.51	10.07	1.24	5.01	987.72	474.10	333.17	32.17	148.29
	Indonesia	69.18	37.24	16.09	8.33	7.52	31.93	17.98	6.96	2.43	4.55	1183.49	666.67	267.31	81.63	167.89
	Italy	128.31	85.13	8.94	25.48	8.76	27.94	18.13	2.21	2.38	5.22	804.93	528.64	69.90	67.30	139.09
	Japan	101.09	68.17	13.29	11.98	7.66	20.40	11.32	3.00	2.29	3.80	668.14	379.45	106.01	66.92	115.77
	Republic of Korea	58.12	37.88	9.57	4.89	5.78	12.85	6.41	2.52	0.99	2.93	425.90	232.46	78.75	28.05	86.63
	Mexico	63.66	32.24	16.45	7.32	7.65	25.85	12.16	7.04	1.75	4.90	807.22	375.29	230.57	50.34	151.02
	Russian Federation	105.72	49.06	17.29	30.14	9.24	30.25	15.08	5.40	4.37	5.40	938.63	451.74	202.10	123.94	160.86
	Saudi Arabia	65.85	49.36	4.88	5.25	6.36	25.69	17.86	2.20	1.28	4.35	705.03	494.91	63.06	32.80	114.25
	South Africa	101.49	41.88	47.54	5.36	6.72	55.08	23.17	24.48	2.42	5.01	1745.81	706.56	834.46	60.01	144.79
	Türkiye	63.04	41.74	4.45	9.49	7.36	21.54	12.87	1.82	2.16	4.69	648.48	405.60	54.82	55.89	132.18
	United Kingdom	139.34	89.50	14.73	23.66	11.45	34.34	20.83	2.60	3.83	7.08	936.13	578.02	90.80	92.50	174.80
	United States of America	149.98	92.47	14.60	34.14	8.76	28.59	16.77	2.65	3.93	5.24	845.82	505.17	95.20	109.31	136.14
	European Union	122.83	83.31	9.94	20.19	9.39	31.05	19.03	3.18	2.97	5.87	876.99	547.56	101.18	75.50	152.75
	African Union	112.42	53.10	45.89	5.31	8.11	56.72	27.32	21.86	2.15	5.39	2045.85	962.08	841.44	61.16	181.17
European Union	Austria	112.97	77.33	8.51	19.00	8.13	28.18	17.73	2.48	2.76	5.20	755.20	482.54	77.15	67.63	127.88
	Belgium	117.74	83.23	7.85	17.99	8.68	30.57	19.62	2.37	2.78	5.80	824.95	542.56	74.26	67.22	140.90
	Bulgaria	97.96	51.74	16.73	19.42	10.07	33.72	17.84	5.58	4.09	6.21	1016.24	523.15	191.56	111.07	190.45
	Croatia	102.69	62.23	9.26	21.11	10.08	30.23	16.53	3.18	3.81	6.71	874.05	489.92	104.02	96.58	183.52
	Cyprus	128.70	89.41	9.06	20.43	9.80	35.44	22.27	2.98	3.48	6.72	901.13	581.95	83.85	78.22	157.10
	Czechia	100.53	59.82	9.48	20.37	10.86	29.67	16.22	3.66	3.50	6.29	828.24	450.91	114.16	89.78	173.40
	Denmark	123.35	84.56	9.53	19.65	9.61	34.65	22.16	3.09	2.67	6.73	883.74	568.44	90.13	66.32	158.85
	Estonia	97.75	56.83	11.59	19.96	9.37	27.64	14.57	4.37	2.99	5.71	786.35	411.72	135.58	79.55	159.50
	Finland	130.96	92.28	6.07	23.51	9.10	27.74	17.39	1.48	3.60	5.27	761.79	499.18	47.81	86.79	128.01
	Greece	115.14	79.38	7.82	19.11	8.84	31.33	20.38	2.57	2.87	5.50	858.45	557.03	79.18	74.27	147.97
	Hungary	102.42	60.67	13.04	19.08	9.63	35.18	19.77	4.91	3.87	6.63	1001.50	554.97	163.20	101.19	182.15
	Ireland	143.24	100.03	8.10	23.44	11.67	33.60	20.51	2.32	3.09	7.68	905.31	572.32	71.80	76.37	184.81
	Latvia	101.09	56.92	10.97	21.07	12.13	34.16	17.76	4.65	3.91	7.84	986.86	512.95	145.71	103.96	224.24
	Lithuania	112.57	58.75	18.63	23.35	11.84	33.88	16.95	5.27	4.11	7.56	1010.79	511.03	173.56	111.62	214.57
	Luxembourg	114.95	79.52	4.84	22.54	8.05	28.73	18.48	1.60	3.32	5.33	735.57	483.56	45.37	78.96	127.69
	Malta	119.86	85.22	4.80	19.90	9.94	32.33	21.00	1.53	3.33	6.47	876.84	587.64	46.23	79.51	163.47
	Netherlands	121.28	87.56	7.53	17.33	8.86	31.82	20.31	1.88	3.19	6.44	845.13	561.03	60.51	74.01	149.57
	Poland	93.62	53.50	9.66	19.14	11.32	36.30	19.10	4.54	4.58	8.08	980.12	518.96	136.69	106.91	217.56
	Portugal	139.98	94.33	13.80	25.57	6.27	25.77	16.35	2.91	2.98	3.52	782.57	516.33	96.23	77.97	92.04
	Romania	97.82	52.32	23.45	13.36	8.69	36.88	18.49	9.49	2.98	5.92	1099.94	535.22	314.89	79.41	170.42
	Slovakia	104.66	58.41	13.86	22.65	9.73	36.68	20.11	5.23	4.83	6.51	1031.15	550.41	171.93	123.88	184.94
	Slovenia	109.41	71.93	7.46	22.31	7.70	29.18	18.03	2.42	3.45	5.27	781.11	479.49	74.84	86.94	139.83
	Spain	101.86	64.97	7.85	21.44	7.60	23.36	13.90	2.12	2.79	4.55	679.77	418.44	68.06	71.20	122.07
	Sweden	109.38	73.34	6.58	21.86	7.60	24.98	14.50	2.50	2.73	5.24	661.54	402.85	68.43	65.93	124.32
African Union	Burundi	122.92	33.42	77.03	4.13	8.34	62.77	20.08	35.50	1.89	5.29	2531.06	744.92	1523.25	61.33	201.57
	Cameroon	124.41	65.13	42.99	7.06	9.24	70.29	37.96	22.31	3.41	6.61	2365.88	1268.18	797.41	90.42	209.86
	Central African Republic	102.94	36.72	58.44	2.40	5.38	61.13	23.34	32.77	1.28	3.75	2378.07	916.19	1285.10	40.21	136.57
	Chad	93.78	33.01	51.49	4.90	4.37	55.85	21.64	28.22	2.79	3.21	1951.91	731.66	1042.93	73.62	103.70
	Congo	128.42	73.73	40.02	3.61	11.06	70.99	40.69	21.13	1.60	7.58	2571.24	1502.19	765.60	46.97	256.48
	Democratic Republic of the Congo	164.17	73.02	76.33	4.22	10.60	85.48	39.90	36.94	1.84	6.80	3354.93	1584.71	1462.07	58.24	249.91
	Equatorial Guinea	204.13	107.01	75.55	6.03	15.54	99.00	51.26	35.58	2.19	9.97	3695.74	1951.06	1329.24	67.19	348.25
	Gabon	155.92	88.82	49.09	4.78	13.23	77.71	43.88	23.45	1.83	8.55	2892.25	1660.64	878.44	55.60	297.57
	Sao Tome and Principe	120.51	49.11	52.19	10.30	8.91	59.34	25.09	23.68	4.41	6.16	2056.88	857.38	887.07	113.47	198.97
	Comoros	36.84	14.09	16.92	2.30	3.53	21.17	8.63	9.01	1.02	2.51	721.91	277.68	331.31	29.80	83.11
	Djibouti	111.37	36.87	60.28	5.68	8.54	54.53	20.62	26.22	2.36	5.34	2120.89	747.97	1100.08	74.75	198.09
	Eritrea	157.36	45.68	93.60	6.24	11.84	77.65	27.00	40.69	2.82	7.15	3249.41	1051.21	1816.26	95.69	286.25
	Ethiopia	175.68	54.82	97.93	7.30	15.63	88.50	31.32	44.16	3.05	9.97	3296.70	1062.62	1782.25	93.58	358.25
	Kenya	111.04	49.73	48.02	4.84	8.44	53.31	25.17	20.94	1.92	5.28	2037.98	943.30	847.38	58.88	188.42
	Madagascar	147.44	48.55	80.22	6.96	11.71	80.54	30.23	39.03	3.29	7.99	2947.62	1015.25	1557.62	97.52	277.24
	Mauritius	101.58	59.46	15.37	17.58	9.17	40.70	24.44	6.35	4.14	5.77	1279.19	766.43	208.64	121.29	182.82
	Rwanda	148.51	54.33	74.94	5.84	13.39	75.67	31.21	33.40	2.46	8.61	2837.58	1084.98	1367.43	76.60	308.57
	Seychelles	109.94	51.46	36.18	10.00	12.30	46.55	21.58	14.91	2.51	7.56	1535.88	711.43	503.81	74.77	245.87
	Somalia	53.94	11.15	38.25	1.87	2.67	29.96	7.55	19.58	1.02	1.81	1196.65	269.77	827.90	31.70	67.27
	South Sudan	161.04	43.51	99.27	6.04	12.21	84.90	27.13	47.05	2.92	7.81	3438.13	1015.49	2026.36	95.48	300.80
	Sudan	47.49	31.31	7.11	5.15	3.92	21.04	13.45	3.42	1.66	2.51	708.47	470.01	110.93	47.07	80.46
	United Republic of Tanzania	115.66	26.15	76.21	6.27	7.03	67.14	15.94	42.98	3.01	5.22	2191.13	503.05	1448.28	81.05	158.76
	Uganda	163.30	68.73	69.81	8.82	15.94	78.02	35.45	29.21	3.54	9.83	3063.98	1364.32	1228.73	110.31	360.62
	Algeria	61.66	40.66	14.02	3.03	3.96	20.96	12.83	5.04	0.70	2.39	724.41	452.67	177.31	19.78	74.65
	Egypt	69.14	53.52	5.32	5.20	5.10	30.54	22.79	2.66	1.57	3.53	899.83	679.57	78.56	41.55	100.14
	Libya	67.90	41.13	11.41	8.12	7.24	25.66	14.59	4.45	2.09	4.52	864.20	506.78	155.68	59.43	142.31
	Mauritania	130.21	66.17	46.44	9.04	8.56	65.98	34.26	22.09	3.77	5.87	2246.19	1164.27	793.66	100.82	187.44
	Morocco	93.48	58.03	22.06	4.21	9.18	38.67	22.50	9.04	1.26	5.87	1363.17	818.93	323.01	35.31	185.93
	Tunisia	113.04	88.33	7.13	12.43	5.14	36.13	27.17	3.09	2.64	3.23	1117.14	858.55	91.38	72.84	94.37
	Angola	91.64	44.09	38.27	2.66	6.62	48.84	24.40	18.93	1.18	4.32	1870.75	946.18	733.10	36.42	155.05
	Botswana	95.52	34.66	51.01	5.47	4.38	43.91	17.15	21.76	2.22	2.78	1624.55	626.98	840.51	61.78	95.28
	Eswatini	209.77	37.68	157.63	8.27	6.19	97.06	20.35	69.44	3.55	3.71	4099.40	836.63	2999.15	116.03	147.59
	Lesotho	112.07	28.47	73.28	4.43	5.90	69.70	19.06	43.57	2.49	4.58	2314.04	599.67	1511.84	64.00	138.53
	Malawi	190.37	41.76	135.87	3.23	9.50	91.65	24.21	60.03	1.40	6.00	3776.66	904.63	2598.73	45.76	227.53
	Mozambique	57.50	12.83	41.12	1.47	2.09	33.28	8.99	21.94	0.80	1.55	1158.57	265.82	822.36	21.63	48.77
	Namibia	117.73	62.97	44.31	4.20	6.25	55.87	31.97	18.35	1.70	3.85	2198.58	1244.44	761.15	51.49	141.51
	Zambia	189.93	65.75	112.59	4.54	7.05	101.01	38.41	55.85	2.02	4.73	3745.29	1355.04	2163.81	61.17	165.27
	Zimbabwe	54.43	23.24	24.38	2.17	4.63	33.54	14.93	13.95	1.18	3.48	1112.36	481.41	492.44	30.59	107.92
	Benin	58.54	33.33	18.93	3.13	3.16	30.84	18.50	8.77	1.43	2.13	1129.85	674.03	343.35	40.52	71.95
	Burkina Faso	78.96	39.63	30.57	4.25	4.51	48.19	24.96	17.47	2.33	3.43	1546.67	798.59	588.54	57.63	101.91
	Cabo Verde	87.06	41.80	33.93	7.82	3.52	39.91	18.99	15.57	3.03	2.32	1271.01	612.99	512.69	71.95	73.37
	Côte d'Ivoire	135.76	86.65	33.67	6.01	9.44	73.45	47.29	17.22	2.67	6.27	2712.85	1776.78	639.12	78.37	218.58
	Gambia	65.45	34.82	25.80	2.38	2.45	35.66	20.50	12.33	1.13	1.70	1265.67	701.74	475.96	31.58	56.38
	Ghana	95.07	45.28	31.95	9.22	8.62	49.44	24.14	15.34	4.13	5.83	1769.43	881.39	578.51	112.75	196.78
	Guinea	106.04	33.84	62.28	4.10	5.82	58.02	20.25	31.72	2.02	4.03	2170.20	738.82	1234.64	58.42	138.32
	Guinea-Bissau	105.59	47.31	46.26	5.25	6.77	62.77	30.48	24.54	2.93	4.82	2274.43	1087.65	942.29	81.27	163.21
	Liberia	134.36	67.08	51.82	6.34	9.11	70.71	37.01	24.75	2.86	6.09	2603.32	1353.21	958.13	81.87	210.11
	Mali	135.93	67.96	53.96	6.68	7.33	73.97	38.74	27.02	3.20	5.00	2778.76	1464.39	1049.91	92.41	172.05
	Niger	70.02	39.74	14.31	4.19	11.77	41.83	24.10	7.18	2.17	8.38	1442.04	838.16	275.02	58.27	270.59
	Nigeria	118.56	72.11	33.88	4.58	7.98	62.91	38.73	16.61	2.02	5.55	2184.01	1341.22	610.60	55.31	176.88
	Senegal	86.38	41.98	33.55	5.26	5.60	46.74	23.77	16.62	2.46	3.88	1637.69	826.43	616.93	66.96	127.37
	Sierra Leone	95.45	38.68	43.91	5.49	7.37	51.21	22.09	21.47	2.65	5.00	1877.14	805.39	827.28	73.80	170.67
	Togo	102.73	51.34	38.57	5.79	7.02	56.82	29.72	19.47	2.79	4.85	2016.07	1047.39	729.35	76.95	162.38

Fig. S1 Age-standardized incidence, mortality, and DALY rates of women's cancers in 2023 in 98 locations in G20. DALY. Disability-adjusted life year

Group	Location	Age-standardized prevalence rate (1/100,000)					QCI					5-year relative survival				
		Women's cancer	Breast cancer	Cervical cancer	Uterine cancer	Ovarian cancer	Women's cancer	Breast cancer	Cervical cancer	Uterine cancer	Ovarian cancer	Women's cancer	Breast cancer	Cervical cancer	Uterine cancer	Ovarian cancer
G20	Argentina	838.43	565.36	184.53	56.29	32.25	72.10	75.23	72.06	74.45	59.11	60.13	59.53	65.53	71.07	33.24
	Australia	1073.77	829.42	71.77	137.43	35.15	96.30	97.96	93.16	95.72	78.93	78.64	80.70	78.94	87.02	39.52
	Brazil	531.20	347.21	109.69	51.98	22.32	63.49	64.71	61.43	66.70	52.18	60.51	62.37	60.72	68.84	33.65
	Canada	1226.75	873.23	113.48	197.77	42.28	93.61	94.51	97.92	94.78	76.12	78.41	79.63	83.10	86.43	38.62
	China	463.10	316.89	76.52	51.92	17.77	85.16	89.63	74.39	89.38	82.30	73.91	78.63	65.28	83.36	44.32
	France	1483.96	1204.04	62.84	166.62	50.46	95.51	95.61	82.83	94.60	90.25	79.56	82.02	71.35	86.43	45.17
	Germany	1192.96	976.40	52.24	124.25	40.08	88.51	89.07	71.10	91.13	67.31	75.32	77.94	69.43	85.27	37.87
	India	345.74	215.08	88.09	18.07	24.50	44.15	49.21	40.50	53.35	37.60	45.88	47.26	47.91	60.47	27.72
	Indonesia	487.83	309.90	87.70	57.53	32.69	55.84	55.17	54.79	68.28	58.78	53.85	51.71	56.77	70.77	39.49
	Italy	1267.22	952.94	63.87	209.37	41.05	95.91	96.79	88.74	99.94	78.81	78.23	78.71	75.29	90.66	40.41
	Japan	972.02	742.54	96.82	92.44	40.22	95.06	97.73	90.31	86.91	95.52	79.82	83.40	77.43	80.89	50.40
	Republic of Korea	529.98	402.06	63.30	36.18	28.44	91.08	95.55	83.95	84.00	90.00	77.89	83.08	73.71	79.66	49.22
	Mexico	431.34	260.23	88.83	50.91	31.38	62.39	66.00	58.33	76.90	57.07	59.40	62.29	57.21	76.10	35.96
	Russian Federation	871.20	475.82	114.01	238.27	43.10	81.74	82.15	76.63	93.00	76.57	71.39	69.25	68.76	85.51	41.64
	Saudi Arabia	505.45	424.45	24.26	33.53	23.20	71.77	72.69	55.88	74.19	50.07	60.99	63.81	54.94	75.68	31.61
	South Africa	728.58	374.35	292.04	33.88	28.31	53.66	59.30	56.40	54.41	50.46	45.73	44.67	48.50	54.81	25.55
	Türkiye	512.29	385.81	25.65	69.20	31.64	77.09	78.87	65.39	81.79	65.48	65.83	69.18	59.04	77.20	36.31
	United Kingdom	1285.09	939.45	111.27	183.26	51.11	91.18	92.49	96.30	91.64	75.28	75.36	76.73	82.35	83.81	38.19
	United States of America	1445.12	1012.91	112.91	278.43	40.87	96.58	97.55	96.79	97.51	79.03	80.93	81.87	81.82	88.48	40.19
	European Union	1131.93	869.82	63.03	157.79	41.29	90.34	92.15	76.75	93.04	71.77	74.72	77.16	68.04	85.28	37.54
	African Union	709.52	410.26	235.75	31.45	32.05	46.60	49.53	46.52	51.55	47.40	49.54	48.55	52.36	59.57	33.57
European Union	Austria	1068.84	825.73	57.57	149.82	35.71	92.23	93.77	82.49	93.96	71.63	75.06	77.07	70.82	85.48	35.97
	Belgium	1141.03	910.63	52.76	140.92	36.73	92.48	94.43	81.22	92.89	66.27	74.03	76.43	69.77	84.55	33.11
	Bulgaria	779.28	486.46	105.63	142.66	44.53	75.88	78.10	73.92	83.39	66.62	65.58	65.51	66.67	78.97	38.34
	Croatia	900.24	637.11	58.92	161.88	42.32	84.59	88.05	74.45	89.00	61.61	70.56	73.44	65.63	81.97	33.41
	Cyprus	1166.26	912.38	57.65	155.88	40.35	88.94	90.51	77.80	90.76	63.70	72.46	75.09	67.12	82.97	31.49
	Czechia	868.68	600.10	58.05	158.93	51.59	84.33	87.31	69.67	90.56	80.93	70.49	72.89	61.38	82.83	42.10
	Denmark	1146.77	890.36	62.47	155.55	38.39	89.61	91.37	79.08	94.91	60.28	71.91	73.79	67.56	86.42	30.00
	Estonia	844.22	573.49	70.71	157.55	42.48	85.02	88.46	70.26	92.92	73.31	71.73	74.36	62.32	85.04	39.01
	Finland	1253.89	983.16	43.43	184.12	43.19	95.36	96.05	88.86	92.97	84.52	78.81	81.16	75.63	84.67	42.07
	Greece	1090.40	848.25	51.03	151.16	39.96	90.42	91.91	77.82	93.24	72.88	72.79	74.32	67.14	84.97	37.76
	Hungary	843.19	582.42	79.29	142.73	38.74	78.15	81.02	68.83	85.39	55.27	65.65	67.42	62.32	79.74	31.10
	Ireland	1354.67	1062.88	55.52	186.08	50.18	93.30	94.99	83.66	95.45	69.10	76.55	79.50	71.31	86.83	34.24
	Latvia	832.54	557.67	62.53	160.52	51.82	78.47	82.72	62.82	87.76	63.80	66.21	68.79	57.66	81.44	35.38
	Lithuania	936.63	578.60	126.68	180.00	51.35	81.39	84.16	82.61	89.02	65.94	69.90	71.16	71.72	82.40	36.20
	Luxembourg	1098.83	857.12	31.18	176.25	34.28	92.99	94.34	78.18	93.58	68.10	75.00	76.76	66.87	85.28	33.77
	Malta	1109.62	881.04	31.48	154.14	42.96	89.74	90.73	79.19	91.27	68.74	73.03	75.36	68.03	83.28	34.90
	Netherlands	1195.30	973.72	53.99	132.57	35.01	93.15	95.53	88.45	89.37	56.16	73.76	76.80	74.96	81.61	27.38
	Poland	754.81	518.24	52.13	139.80	44.64	74.45	79.64	56.73	82.01	51.62	61.23	64.30	52.96	76.07	28.63
	Portugal	1372.44	1033.30	102.10	206.24	30.80	97.80	97.90	92.93	97.27	87.23	81.59	82.67	78.91	88.33	43.90
	Romania	770.96	499.41	137.03	99.14	35.38	72.85	78.34	64.24	82.86	55.47	62.30	64.65	59.54	77.70	31.92
	Slovakia	839.19	546.38	83.74	168.72	40.35	76.61	78.74	68.66	84.38	58.48	64.95	65.57	62.24	78.68	33.14
	Slovenia	974.58	718.17	48.85	176.15	31.41	88.15	88.90	78.28	92.86	58.66	73.33	74.94	67.52	84.52	31.58
	Spain	989.57	726.38	54.90	172.72	35.56	94.21	96.41	85.54	96.07	78.44	77.07	78.61	72.96	87.00	40.07
	Sweden	1053.17	807.16	40.40	173.95	31.66	94.60	97.30	72.37	96.32	63.24	77.16	80.23	61.99	87.51	31.02
African Union	Burundi	693.94	241.59	395.44	22.85	34.06	35.84	35.70	44.45	37.41	47.25	48.94	39.93	53.91	54.12	36.56
	Cameroon	721.96	451.89	200.68	36.13	33.26	38.70	38.86	39.33	36.63	35.95	43.50	41.71	48.09	51.66	28.51
	Central African Republic	577.83	279.24	265.05	12.50	21.04	28.49	33.72	30.17	27.56	39.64	40.62	36.45	43.93	46.89	30.32
	Chad	497.26	230.72	229.30	22.08	15.17	28.80	31.76	32.21	20.68	30.80	40.44	34.44	45.19	43.22	26.63
	Congo	664.08	464.52	147.33	17.16	35.06	30.54	31.23	24.42	33.80	25.30	44.72	44.82	47.21	55.76	31.51
	Democratic Republic of the Congo	954.12	522.86	365.97	23.43	41.86	37.51	37.59	40.75	41.05	45.27	47.93	45.36	51.60	56.35	35.84
	Equatorial Guinea	929.70	590.84	265.67	27.59	45.61	29.40	29.27	26.42	39.14	22.60	51.50	52.10	52.90	63.67	35.85
	Gabon	981.69	661.32	239.82	28.29	52.27	46.24	46.96	44.48	52.05	47.59	50.16	50.60	52.23	61.83	35.34
	Sao Tome and Principe	680.17	347.16	247.98	53.94	31.08	43.01	45.31	45.30	44.43	35.79	50.76	48.92	54.63	57.16	30.81
	Comoros	231.58	120.18	84.12	13.45	13.83	41.66	49.71	41.23	46.30	43.11	42.53	38.75	46.75	55.61	28.83
	Djibouti	672.93	279.64	323.94	33.66	35.69	43.43	43.60	50.83	47.50	52.36	51.03	44.09	56.51	58.54	37.40
	Eritrea	922.99	339.56	498.39	34.69	50.35	38.37	36.37	48.05	36.87	51.51	50.65	40.89	56.53	54.82	39.62
	Ethiopia	967.43	377.98	487.84	40.66	60.95	38.11	38.67	45.72	43.96	45.60	49.63	42.86	54.91	58.24	36.22
	Kenya	685.64	371.74	252.11	27.66	34.14	45.99	46.34	50.20	48.00	50.91	51.99	49.39	56.40	60.39	37.42
	Madagascar	817.13	337.88	397.71	36.88	44.67	35.03	35.33	42.39	36.43	40.17	45.37	37.74	51.34	52.71	31.79
	Mauritius	778.44	521.85	88.76	128.15	39.67	68.79	68.20	63.17	79.38	62.30	59.93	58.90	58.72	76.43	37.05
	Rwanda	870.38	389.14	392.90	34.02	54.31	40.95	40.09	48.96	46.24	48.76	49.04	42.57	55.44	57.91	35.68
	Seychelles	796.69	461.73	208.81	71.82	54.33	63.33	67.78	62.55	76.98	64.29	57.66	58.07	58.79	74.92	38.56
	Somalia	324.14	95.18	206.86	10.46	11.63	35.73	41.91	43.33	31.41	51.36	44.46	32.28	48.80	45.82	32.27
	South Sudan	883.58	320.75	482.89	31.67	48.27	31.99	33.94	39.65	30.76	43.11	47.28	37.66	52.61	51.62	36.08
	Sudan	346.58	260.17	36.00	33.97	16.43	61.74	61.44	51.20	66.39	58.95	55.69	57.03	51.87	67.84	36.01
	United Republic of Tanzania	613.98	210.60	343.59	34.30	25.48	33.19	47.02	35.08	40.88	35.69	41.95	39.07	43.61	52.06	25.80
	Uganda	1112.87	564.96	418.72	56.29	72.90	50.96	50.83	58.57	53.61	61.08	52.22	48.43	58.16	59.86	38.34
	Algeria	498.74	375.35	84.63	21.45	17.32	74.88	76.42	68.99	79.01	67.87	66.01	68.44	64.08	76.89	39.61
	Egypt	501.18	423.82	25.49	32.93	18.95	64.29	62.77	49.25	67.60	47.96	55.82	57.42	50.09	69.80	30.76
	Libya	526.16	379.49	64.05	53.07	29.55	69.84	73.16	62.82	71.91	58.39	62.21	64.52	61.01	74.19	37.54
	Mauritania	793.41	483.75	228.15	49.77	31.74	46.28	47.10	46.19	47.35	42.11	49.33	48.22	52.45	58.34	31.40
	Morocco	602.16	434.42	109.58	24.66	33.50	57.06	58.19	53.60	62.36	47.04	58.63	61.22	59.04	70.06	36.00
	Tunisia	1080.92	901.19	45.37	108.65	25.70	86.28	84.48	68.73	92.54	77.86	68.04	69.24	56.65	78.77	37.13
	Angola	553.24	332.30	180.53	14.65	25.75	39.30	40.98	39.63	40.62	44.74	46.70	44.65	50.54	55.44	34.67
	Botswana	425.03	196.78	188.29	24.94	15.03	31.26	30.83	33.24	37.24	39.12	54.03	50.53	57.34	59.37	36.64
	Eswatini	1457.92	360.10	1010.88	55.43	31.51	51.24	56.61	59.68	53.04	70.63	53.73	45.98	55.94	57.04	40.09
	Lesotho	518.31	187.53	293.26	18.65	18.87	20.89	30.00	24.05	19.27	22.88	37.80	33.05	40.53	43.67	22.40
	Malawi	1044.88	295.71	693.76	17.78	37.62	36.51	35.90	46.00	39.64	45.30	51.86	42.02	55.82	56.65	36.88
	Mozambique	417.92	118.23	281.07	8.69	9.93	48.82	51.51	58.64	40.10	58.37	42.12	29.91	46.65	45.25	25.79
	Namibia	866.13	534.58	277.32	25.29	28.94	55.16	53.04	61.65	50.91	62.64	52.54	49.23	58.59	59.53	38.34
	Zambia	1067.77	464.43	551.75	24.04	27.54	36.42	37.75	41.36	38.80	43.63	46.82	41.58	50.39	55.59	32.85
	Zimbabwe	346.98	194.66	123.14	11.19	17.99	40.11	46.94	39.90	33.69	41.22	38.38	35.75	42.80	45.69	24.88
	Benin	393.46	263.26	100.27	17.42	12.51	47.26	45.97	49.22	42.70	47.57	47.32	44.49	53.66	54.20	32.43
	Burkina Faso	439.28	277.96	126.83	19.67	14.82	33.35	36.32	29.48	27.27	27.32	38.96	37.01	42.85	45.32	23.84
	Cabo Verde	415.53	252.49	113.67	39.12	10.25	40.14	42.58	30.52	48.31	28.63	54.16	54.57	54.12	61.26	34.04
	Côte d'Ivoire	871.98	642.60	158.96	33.49	36.93	43.26	41.88	39.49	42.96	44.50	45.90	45.42	48.85	55.60	33.56
	Gambia	412.17	258.16	131.75	12.91	9.35	43.10	41.38	46.11	40.07	43.50	45.51	41.14	52.19	52.67	30.39
	Ghana	558.17	329.13	148.42	49.10	31.52	41.39	42.68	41.25	41.62	39.03	48.00	46.69	51.98	55.24	32.37
	Guinea	587.71	247.35	297.35	21.09	21.91	33.78	37.07	38.55	33.53	38.76	45.28	40.17	49.07	50.58	30.72
	Guinea-Bissau	594.89	331.18	214.49	24.46	24.76	31.38	31.22	35.14	21.83	34.77	40.56	35.56	46.97	44.17	28.85
	Liberia	802.11	480.69	252.54	34.11	34.78	40.78	40.27	43.13	40.76	42.58	47.37	44.82	52.25	54.85	33.20
	Mali	797.18	479.53	255.32	34.79	27.54	37.17	36.24	38.80	35.34	39.56	45.59	42.99	49.92	52.11	31.78
	Niger	435.01	297.95	71.76	21.23	44.07	38.71	39.74	43.41	32.70	38.35	40.26	39.36	49.83	48.17	28.83
	Nigeria	775.15	547.74	171.26	25.84	30.31	47.15	46.99	45.63	46.08	42.85	46.94	46.30	50.97	55.98	30.36
	Senegal	520.65	307.32	163.88	28.36	21.09	41.57	42.06	42.96	41.04	41.64	45.89	43.36	50.45	53.28	30.59
	Sierra Leone	565.36	290.69	217.40	28.91	28.36	39.43	41.40	43.07	37.54	42.73	46.35	42.89	51.11	51.79	32.08
	Togo	591.81	358.30	178.33	29.51	25.67	37.39	37.32	38.49	35.65	37.42	44.69	42.11	49.54	51.90	30.92

Fig. S2 Age-standardized prevalence rate, QCI, and 5-year relative survival of women's cancers and 4 types in 2023 in 98 locations in G20. QCI. Quality of care index

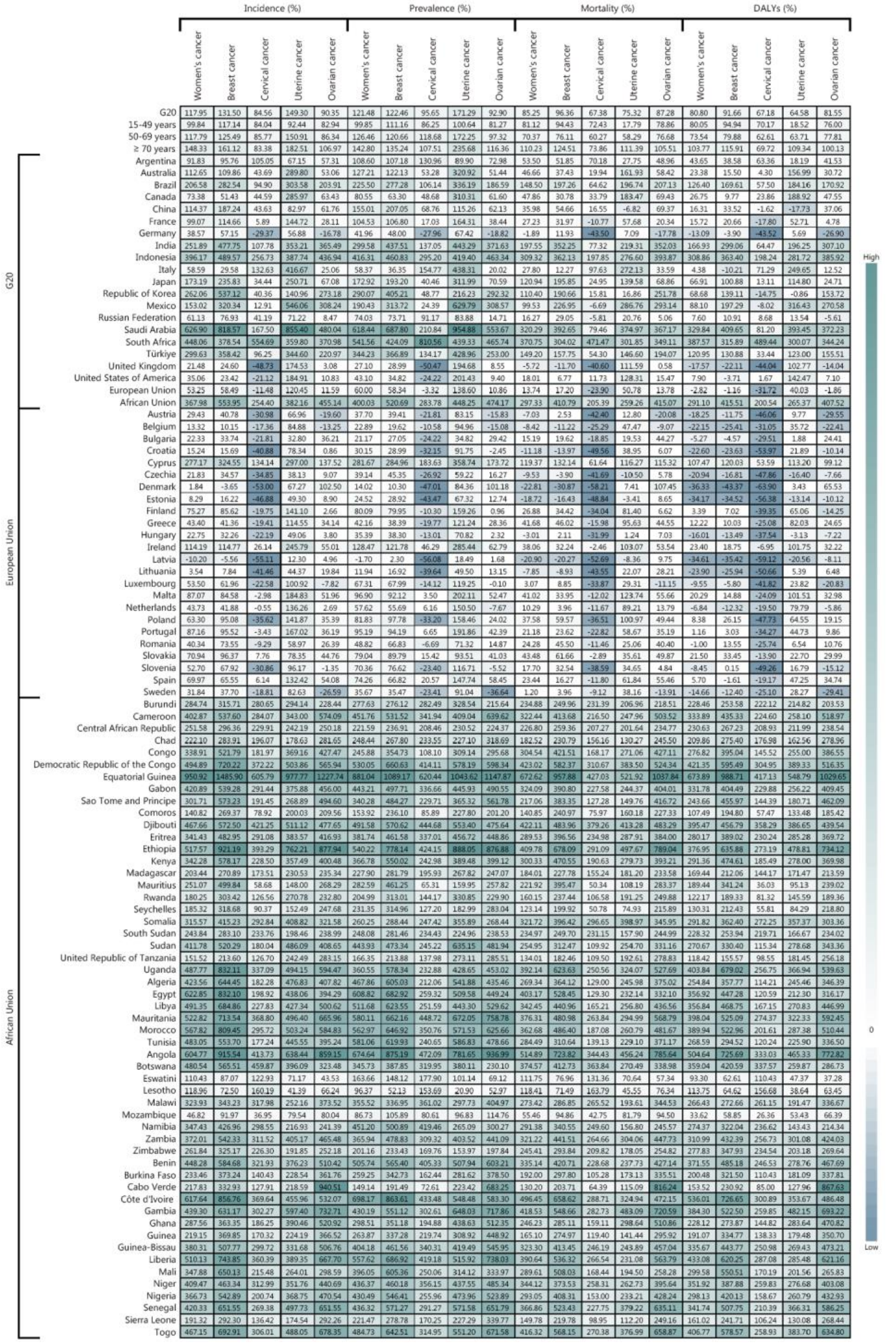

Fig. S3 Percent changes in incidence, prevalence, mortality, and DALYs of women's cancers and 4 types between 1990 and 2023 in G20 and its 98 locations. DALY. Disability-adjusted life year

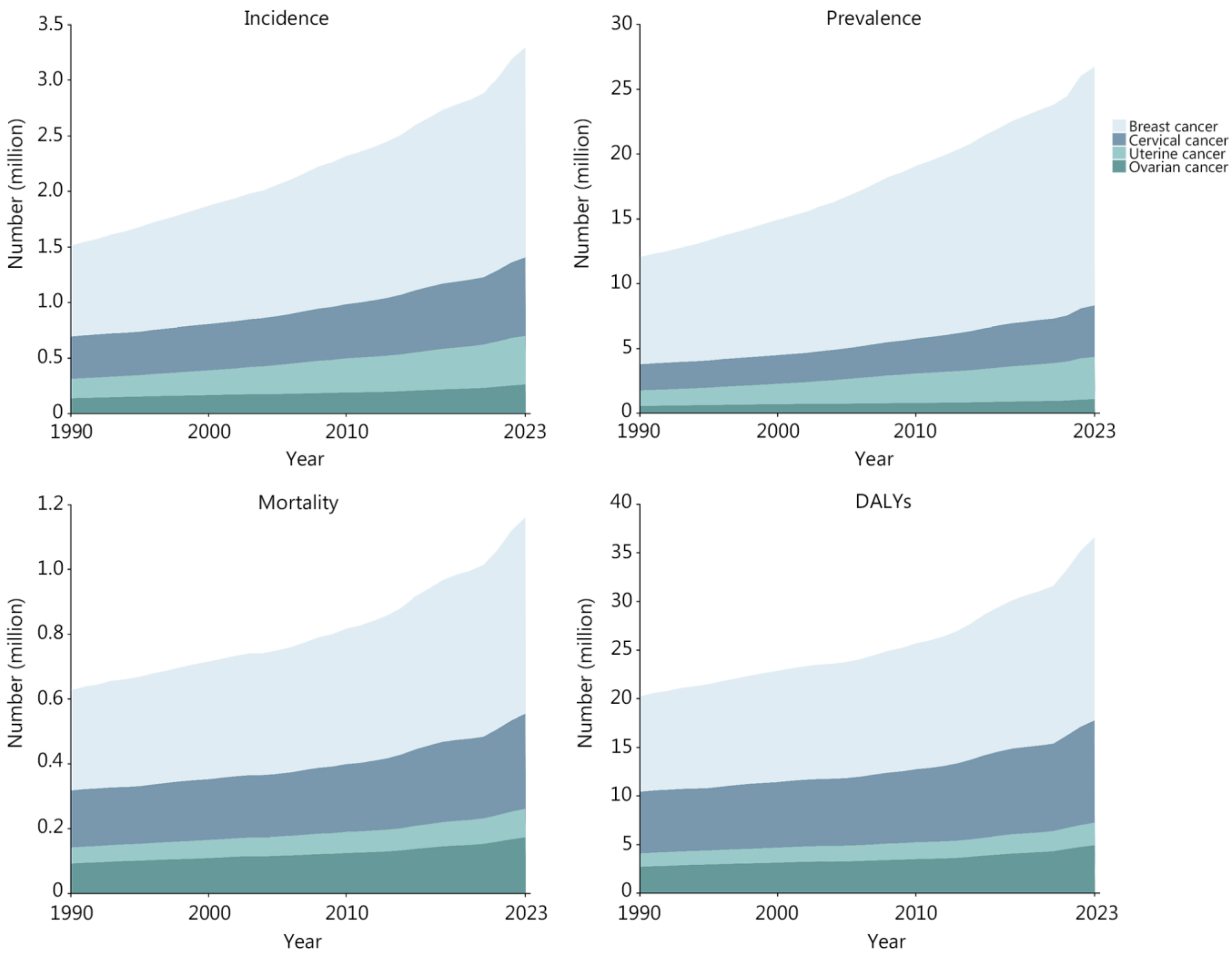

Fig. S4 Incidence, prevalence, mortality, and DALYs of women's cancers by type from 1990 to 2023 in G20. DALY. Disability-adjusted life year

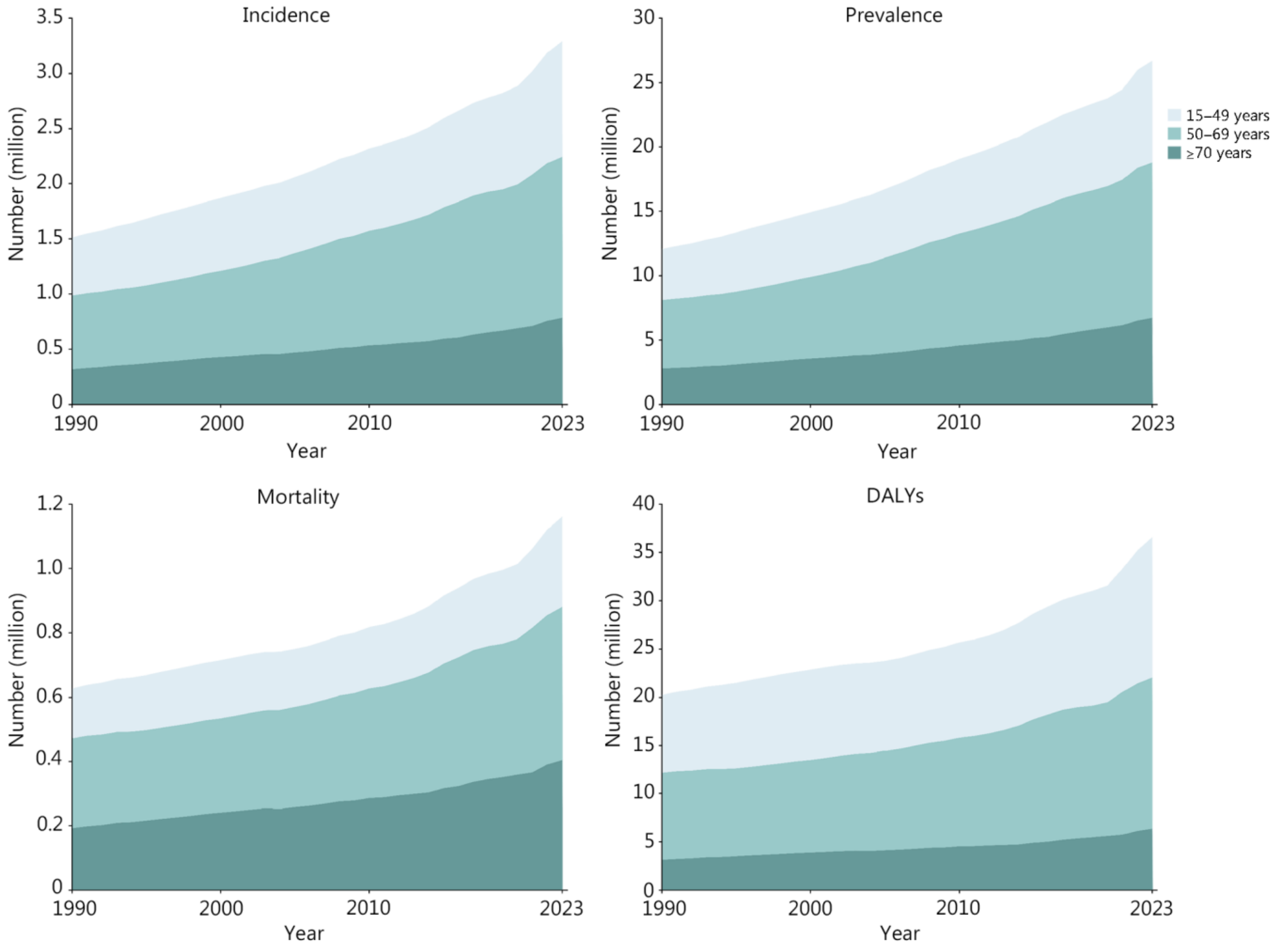

Fig. S5 Incidence, prevalence, mortality, and DALYs of women's cancers by age from 1990 to 2023 in G20. DALY. Disability-adjusted life year

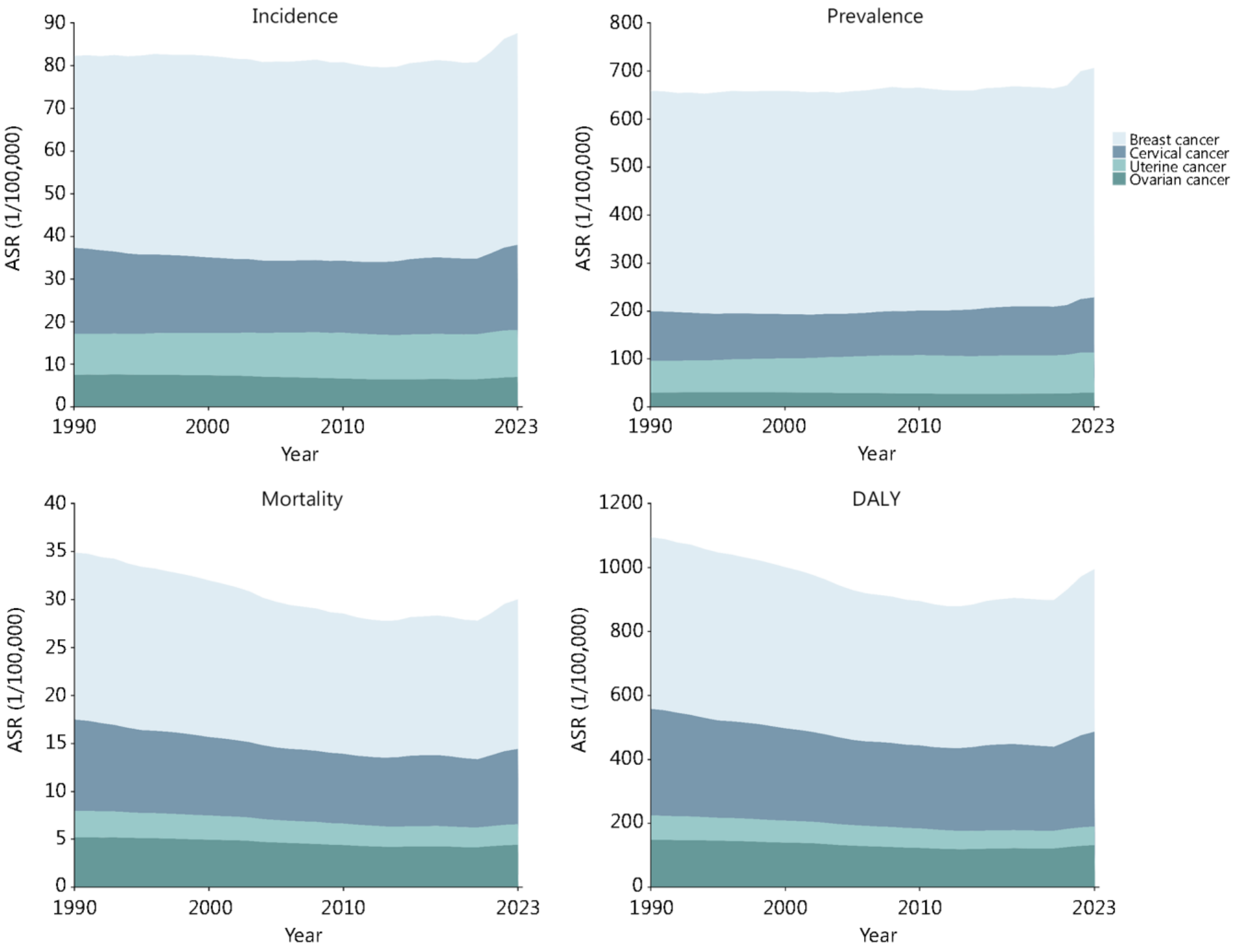

Fig. S6 Age-standardized incidence, prevalence, mortality, and DALY rates of women's cancers by type from 1990 to 2023 in G20. DALY. Disability-adjusted life year; ASR. Age-standardized rate

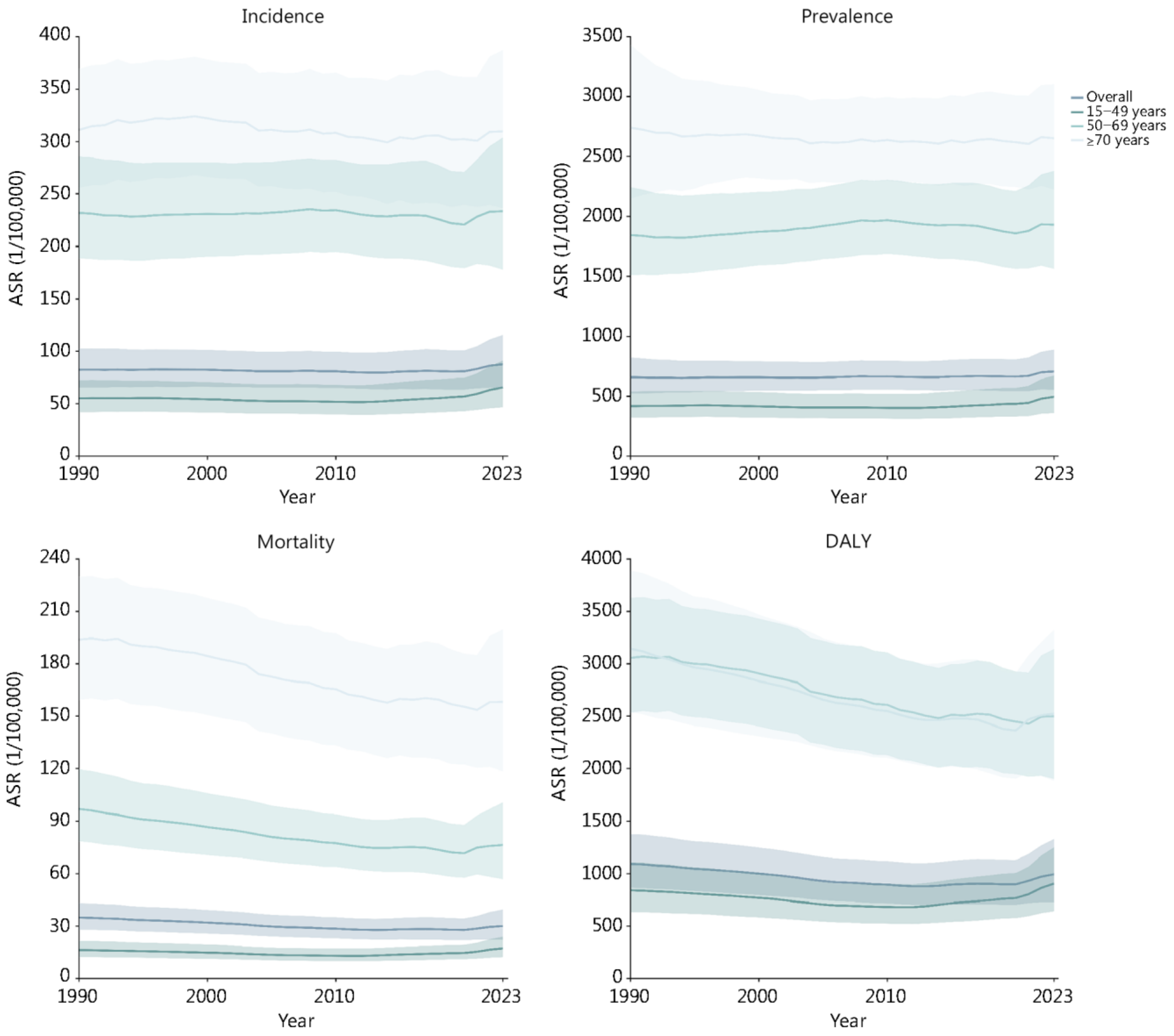

Fig. S7 Age-standardized incidence, prevalence, mortality, and DALY rates of women’s cancers by age from 1990 to 2023 in G20. DALY. Disability-adjusted life year; ASR. Age-standardized rate

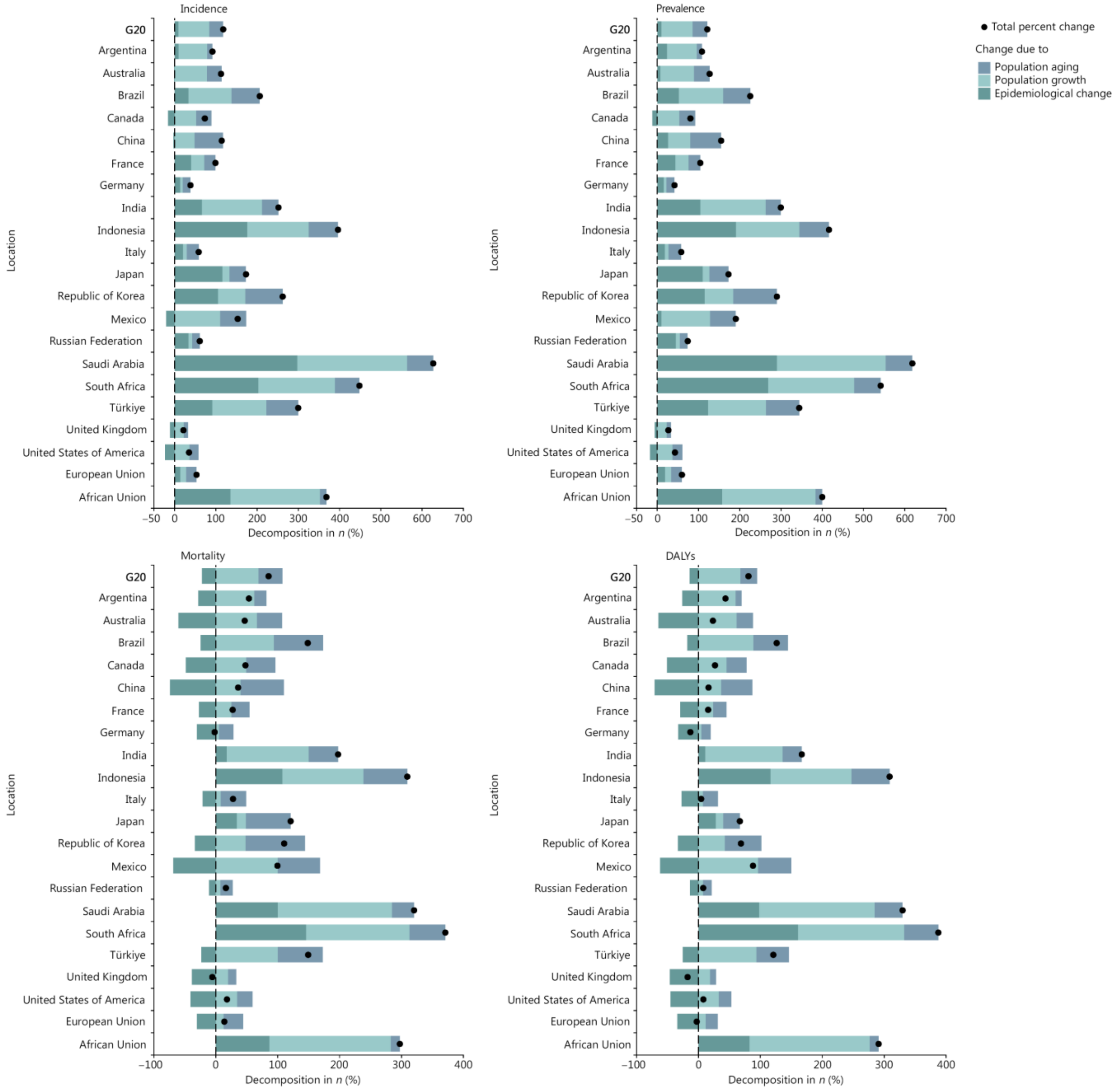

Fig. S8 Decomposition analysis of changes in global incidence, prevalence, mortality, and DALYs of women's cancers in 2023 in G20, compared with 1990. DALY. Disability-adjusted life year
